# Carbon and Nitrogen Metabolism Are Jointly Regulated During Shading in Roots and Leaves of *Camellia Sinensis*

**DOI:** 10.3389/fpls.2022.894840

**Published:** 2022-04-15

**Authors:** Chenyu Shao, Haizhen Jiao, Jiahao Chen, Chenyu Zhang, Jie Liu, Jianjiao Chen, Yunfei Li, Jing Huang, Biao Yang, Zhonghua Liu, Chengwen Shen

**Affiliations:** ^1^Key Laboratory of Tea Science of Ministry of Education, Hunan Agricultural University, Changsha, China; ^2^National Research Center of Engineering and Technology for Utilization of Botanical Functional Ingredients, Hunan Agricultural University, Changsha, China; ^3^Co-innovation Center of Education Ministry for Utilization of Botanical Functional Ingredients, Hunan Agricultural University, Changsha, China; ^4^Tea Research Institution, Chinese Academy of Agricultural Sciences, Hangzhou, China

**Keywords:** *Camellia sinensis*, leaves, roots, shading, metabolism, carbon, nitrogen

## Abstract

Numerous studies have shown that plant shading can promote the quality of green tea. However, the association of shading with metabolic regulation in tea leaves and roots remains unelucidated. Here, the metabolic profiling of two tea cultivars (“Xiangfeicui” and “Jinxuan”) in response to shading and relighting periods during the summer season was performed using non-targeted metabolomics methods. The metabolic pathway analyses revealed that long-term shading remarkably inhibit the sugar metabolism such as glycolysis, galactose metabolism, and pentose phosphate pathway in the leaves and roots of “Xiangfeicui,” and “Jinxuan” were more sensitive to light recovery changes. The lipid metabolism in the leaves and roots of “Xiangfeicui” was promoted by short-term shading, while it was inhibited by long-term shading. In addition, the intensity of the flavonoid metabolites in the leaves and roots of “Jinxuan” were upregulated with a trend of rising first and then decreasing under shading, and five flavonoid synthesis genes showed the same trend (F3H, F3′5′H, DFR, ANS, and ANR). Simultaneously, the amino acids of the nitrogen metabolism in the leaves and roots of the two cultivars were significantly promoted by long-term shading, while the purine and caffeine metabolism was inhibited in the leaves of “Xiangfeicui.” Interestingly, CsGS1.1 and CsTSI, amino acid synthase genes was upregulated in the leaves and roots of two cultivars. These results indicated that shading could participate in carbon and nitrogen metabolic regulation of both leaf and root, and root metabolism could have a positive association with leaf metabolism to promote the shaded tea quality.

## Introduction

Green tea, a type of unfermented tea rich in secondary metabolites, is valued for its “umami” flavor and sweet taste and is known for its antibacterial, anti-obesity, antioxidant, and anticancer activity (Prasanth et al., 2019). The annual tea sales exceed $43 billion globally, more than $11 billion of which is accounted for by green tea (Hu et al., 2018). High-quality green tea is primarily dependent on the contents of the initial metabolite, which differs markedly among cultivars and is strongly influenced by external environmental factors (Pongsuwan et al., 2008; Tang et al., 2021). Secondary metabolites including amino acids, flavonoids, lipids, and terpenoids vary significantly in fresh leaves of different tea cultivars (Maritim et al., 2021). In the same cultivar, the quality and value of tea vary considerably with the harvest season (Dai et al., 2015). Due to the distinct variations in the day length, rainfall, sunlight, and/or temperature between spring, summer and autumn, the chemical composition of tea is dramatically different in different seasons (Nowogrodzki, 2019; Wang et al., 2021). The polyphenol/amino acid ratio is inversely correlated with green tea quality, where a lower ratio makes tea more brisk but less bitter (Guo et al., 2021). Therefore, as summer and autumn tea, which are harvested in warm months (between June and October), possess a high ratio, comparatively few fresh leaves are collected because of the resultant bitterness (Zhang Q. et al., 2020). Therefore, it is necessary to develop strategies for improving the quality of summer and autumn tea.

High light intensity and temperature are essential ecological factors that accelerate flavonoid biosynthesis, tricarboxylic acid (TCA) cycle, and photorespiration in summer tea (Liu et al., 2016). Shading treatment is an effective measure for controlling the amount of sunlight to modify major quality-related metabolites in tea leaves and improve tea flavor (Ku et al., 2010; Ji et al., 2018; Li et al., 2020). In this traditional cultivation method, tea plants are covered with shading nets when new shoots emerge until they are harvested after 14 and 30 days (Sano et al., 2018). During this shading period, the contents of chlorophylls and free amino acids such as theanine are increased, and that of catechins is decreased (Ji et al., 2018). Certain studies have showed that most enzymes controlling amino acid biosynthesis are downregulated in response to shading and the proteolysis of chloroplast proteins results in the accumulation of free amino acids (Chen et al., 2017). Shading improves tea quality by inhibiting the expression of genes regulating flavonoid biosynthesis such as *PAL, 4CL*, and *CHS* (Liu et al., 2018). Shading also influences metabolism in tea roots. Alterations in exogenous nitrogen modulated the genes encoding amino acid biosynthesis in the roots including CsGDH, CsAlaDC, CsAspAT, and others (Yang et al., 2020). However, few studies have investigated the metabolic changes in shaded leaves and roots. Shading may negatively impact tea yield as it forces the plants to grow under low light for extended periods. Recurring artificial shading affects leaf photosynthesis and stomatal transpiration (Yamashita et al., 2020). The sudden onset of intense light after prolonged shading may induce oxidative stress in tea plants (Sano et al., 2020). Hence, the optimal time to pick fresh high-quality tea leaves under shading has not yet been established.

Metabolomic analysis, widely used on tea plants, is a technology used extensively for the comprehensive profiling and comparison of metabolites. Metabolomic approaches including ^1^H NMR, gas chromatography-mass spectrometry (GC-MS), and ultra-performance liquid chromatography combined with time of flight mass spectrometry (UPLC-TOF-MS/MS) have been widely used to explore metabolite accumulation in tea plants (Zhang et al., 2014; Ji et al., 2018; Li et al., 2020). However, the changes in metabolites relating to the carbon and nitrogen metabolism in the leaves and roots of different tea cultivars over different shading periods are unclear. Therefore, in the present study, “Xiangfeicui” and “Jinxuan” were selected for shading (0, 4, and 12 days) and light recovery (4 days). UHPLC-Q-TOF-MS/MS was used to explore the dynamic changes that occur in shaded tea leaves and roots. The aim of this study is not only to provide a framework for better understanding the regulation of leaf and root metabolites in tea cultivars under different shading conditions, but also propose strategies for shade adaptability of different tea cultivars to effectively improve the quality of green tea in the summer and autumn.

## Materials and Methods

### Plant Materials and Experimental Design

The tea plant [*Camellia sinensis* (L.) O. Kuntze cv. “Xiangfeicui” (XFC) and “Jinxuan” (JX)] were cultivated at the experimental tea farm of the Hunan Tea Research Institute in Changsha, China (113.21°E, 28.28°N). They grew for 12 days under 95% shade created by covering the plants with black high-density polyethylene netting, and the net was removed to allow light recovery for 4 days. In 2020, one bud and two leaves were plucked on August 10, 14, 22, and 26, respectively. Light intensity, air temperature and relative humidity, and soil temperature and humidity were measured at 14:00 daily. The samples were washed with ultrapure water and divided into three parts. The first lot comprised fresh leaves used to measure chlorophyll and carotenoid content. The second lot consisted of leaves that were quickly fixed with liquid nitrogen and stored in a −80°C refrigerator for evaluating biochemical composition. The third lot was processed into green tea.

A pot experiment was conducted on the same shading treatment. Each pot contained three healthy 1-year tea plants and 4.5 kg yellow-red soil, and there were six pots per treatment. The dimensions of each pot were top outer diameter: 26.5 cm, top inner diameter: 23 cm, base diameter: 18 cm, and height: 25 cm. There were four drainage holes in the base, and trays were placed under the pots. New shoots could be used for shading when they reached the one terminal bud/two young leaf stage. In 2020, one bud and two leaves and the white absorption roots (Supplementary Figure 1) were collected on September 1, 5, 13, and 17, respectively. The main root lengths and numbers of lateral roots were measured at four sampling points. The samples were washed with ultrapure water and divided into two parts. The first was quickly fixed with liquid nitrogen and stored in a −80°C refrigerator until metabolic analyses (six biological replicates). The second was examined under transmission electron microscopy (fresh leaves).

### Sensory Evaluation

Green tea was processed by one professional tea processor (Supplementary Figure 2). Fresh tea samples were first fixed in an electric frying pot (6CST-70; Xiangfeng Machinery Ltd., Changsha, China) maintained at 320°C using an infrared thermometer (TASI-8601; Suzhou TASI Electronics Ltd., Suzhou, China). After 2 min, they were removed and set on a bamboo plate to cool for 10 min and mechanically rolled for 10 min with a rolling machine (6CD-280; Xiangfeng Machinery Ltd., Changsha, China). The leaves were dried at 220°C in the pot until their moisture content was ~50% and mechanically rolled for 10 min. The leaves were dried in the pot again at 120°C until their moisture content was ~20%. The leaves were then machine-dried at 80°C in a tea dryer (6CHBZ-20; Xiangfeng Machinery Ltd., Changsha, China) until their moisture content was ~5%.

Sensory evaluation was scored independently by 5 professional tea tasters on a 100-point scale, with the appearance of dry tea accounting for 25%, brew color for 10%, aroma for 25%, taste for 30%, and infused leaf for 10% of the scale. All members of the tea assessment group are professional teachers from Hunan Agricultural University. Dry tea (3 g) was brewed with boiling water (150 mL) for 4 min. After brewing, brew color, aroma, taste, and infused leaves were evaluated (Supplementary Figure 3). Scoring rules (Supplementary Table 1) were established according to the National Standard of China (GB/T23776-2018) (Zhou et al., 2020).

### Catechin, Caffeine, and Theanine Content

Catechin, caffeine, and theanine contents were determined using a high-performance liquid chromatography (HPLC) system (Waters 590; Waters Corp., Milford, MA) equipped with a Hypersil ODS 2 C18 column (5 mL, 4.6 × 250 mm, 35 μm) at 280 nm, by referring to the national standards (GB/T 8313–2018, GB/T 8314–2013). Solvents A (2% acetic acid) and B (acetonitrile) were run in a linear gradient from 93 to 55% for 20 min, after which the flow rate was maintained at 1.4 mL/min for 5 min. They were quantitatively determined by comparing the peak areas of samples with a known standard (Sigma-Aldrich, USA). The amino acid content was determined following the methods described previously (Li Z. X. et al., 2016). The amino acid composition was measured by HPLC using an AccQ Tag column (3.9 × 150 mm) (Waters 600; Waters Corp., Milford, MA, USA) and a fluorescence detector, after derivatization with AccQ Fluor Reagent Kit following the manufacturer's protocol.

### Photosynthetic Pigments and Biochemical Components

A digital lux meter (TES 1332; Olympus Imaging 95 Corp., Tokyo, Japan) measured light intensity. A SPAD chlorophyll meter (Konica Minolta Inc., Osaka, Japan) measured leaf SPAD indicating the relative chlorophyll content. The ethanol method was used to determine chlorophyll A and B and carotenoid content in fresh leaves (Zhang C. et al., 2020). State Standards of China Nos. GB/T8313-2008 and GB/T8314-2013 were used to analyze tea leaf polyphenolic and free amino acid content. The foregoing measurements were made within 1 week after sampling.

### Transmission Electron Microscopy Analysis (TEM)

Samples were prepared for TEM as follows (Li N. et al., 2016). Briefly, fresh leaves were sliced into 1-mm^2^ sections, fixed with 2.5% (v/v) glutaraldehyde at 20°C for 2 h, and rinsed thrice with phosphate-buffered solution (pH 7.4) for 15 min each time. The leaf cells were post-fixed with 1% (v/v) OsO_4_ at 20°C for 5 h and dehydrated in an ethanol concentration gradient series [30, 50, 70, 80, 90, 95, and 100% (v/v) for 1 h at each concentration]. The samples were then embedded in 100% Epon-812 and polymerized in an oven at 60°C for 48 h. The embedded samples were cut into ultrathin sections (60–80 nm) with an ultramicrotome (EM UC7; Leica Microsystems Inc., Mannheim, Germany) and stained with uranyl acetate and lead citrate for 30 min. Images were acquired and analyses were made using TEM (HT7700; Hitachi Ltd., Tokyo, Japan).

### UHPLC-Q-TOF MS for Metabolomics

#### Extraction Procedure

Tea samples (80 mg) were quickly frozen in liquid nitrogen and pulverized with a mortar and pestle. One thousand microliters methanol/acetonitrile/H_2_O (2:2:1, v/v/v) mixture were added for metabolite extraction. The mixture was centrifuged at 14,000 × g and 4°C for 15 min, and the supernatant was dried in a vacuum centrifuge. For the LC-MS analysis, the samples were re-dissolved in 100 μL acetonitrile/water (1:1, v/v).

#### LC-MS/MS Analysis

The analyses were performed in the UHPLC (1290 Infinity LC; Agilent Technologies, Santa Clara, CA, USA) coupled to a quadrupole time-of-flight (AB Sciex TripleTOF 6600; AB Sciex, Framingham, MA, USA) at Shanghai Applied Protein Technology Co. Ltd., Shanghai, China.

For hydrophilic interaction liquid chromatography separation, the samples were analyzed with a 2.1 × 100 mm ACQUITY UPLC BEH 1.7 μm column (Waters Corp., Dublin, Ireland). In ESI positive and ESI negative modes, mobile phase A consisted of 25 mM ammonium acetate and 25 mM ammonium hydroxide in water while mobile phase B consisted of acetonitrile. The gradient was 85% B for 1 min, linear reduction to 65% over 11 min, reduction to 40% in 0.1 min, holding for 4 min, and increasing to 85% in 0.1 min. There was a 5-min re-equilibration period.

For reverse-phase liquid chromatography separation, a 2.1 × 100 mm ACQUITY UPLC HSS T3 1.8 μm column (Waters Corp., Dublin, Ireland) was used. In ESI positive mode, mobile phase A was water plus 0.1% (v/v) formic acid while mobile phase B was acetonitrile plus 0.1% (v/v) formic acid. In ESI negative mode, mobile phase A was 0.5 mM ammonium fluoride in water while mobile phase B was acetonitrile. The gradient was 1% B for 1.5 min, linear increase to 99% over 11.5 min, holding for 3.5 min, and reduction to 1% in 0.1 min. There was a 3.4-min re-equilibration period. The flow rate was 0.3 mL/min, the column temperature was maintained at 25°C, and the aliquot injection volume was 2 μL.

The ESI source conditions were as follows: Ion Source Gas1 (Gas1), 60; Ion Source Gas2 (Gas2), 60; curtain gas (Vinocur and Altman, 2005), 30; source temperature, 600°C; and IonSpray Voltage Floating, ±5,500 V. In MS-only acquisition, the instrument was set to acquire over the m/z range 60–1,000 Da and the TOF MS scan accumulation time was set to 0.20 s/spectrum. In auto-MS/MS acquisition, the instrument was set to acquire over the m/z range 25–1,000 Da and the product ion scan accumulation time was set to 0.05 s/spectrum. The product ion scan was performed using information-dependent acquisition in high-sensitivity mode. The parameters were as follows: collision energy, 35 ± 15 eV; declustering potential, 60 V (+) and −60 V (–); exclusion of isotopes within 4 Da; and 10 candidate ions to monitor per cycle.

#### Data Processing and Analysis

Raw MS data (wiff.scan files) were converted to MzXML files with Proteo Wizard MS Convert before being imported into XCMS software. Peaks were selected using the following parameters: centWave m/z = 25 ppm; peakwidth = c (10, 60); and prefilter = c (10, 100). Peaks were grouped using the following parameters: bw = 5; mzwid = 0.025; and minfrac = 0.5. Collection of Algorithms of MEtabolite pRofile Annotation (CAMERA) was used to annotate isotopes and adducts. For the extracted ions, only variables with >50% non-zero measurements values in ≥1 group were conserved. Metabolites were identified by comparing m/z accuracy (<25 ppm) and using MS/MS spectra with an in-house database built using available authentic standards.

After normalization to total peak intensity, the processed data were analyzed in the R package ropls (R Core Team, Vienna, Austria) and subjected to multivariate Pareto-scaled principal component analysis (PCA) and orthogonal partial least-squares discriminant analysis (OPLS-DA). Seven-fold cross-validation and response permutation tests were run to evaluate model robustness. The variable importance in the projection (VIP) value of each variable in the OPLS-DA model was calculated to evaluate its contribution to the classification. Metabolites with VIP >1 were subjected to a univariate-level Student's *t*-test to establish the significance of each metabolite.

### Quantitative Real-Time Polymerase Chain Reaction

Total RNA was extracted from the leaves and roots with the RNA extraction kit (Tiangen, Beijing, China), and complementary DNA (cDNA) was synthesized with a PrimeScript^TM^ RT Reagent kit (Takara, Dalian, China). Supplementary Table 2 lists the primer pairs used for qRT-PCR; glyceraldehyde 3-phosphate dehydrogenase (GAPDH, GenBank accession No. GE651107) was the reference gene. The thermal cycling protocol followed the manufacturer's instructions. Three independent biological replicates of each reaction were conducted, and the relative transcript levels of target genes were calculated against those of GAPDH by the 2^−ΔΔCT^ method.

### Statistical Analyses

Least significant difference (LSD), Duncan's multiple range, and Student's *t*-tests were performed in SPSS v. 25.0 (SPSS Inc., Chicago, IL, USA) to evaluate differences among treatment means. *P* < 0.05 was considered statistically significant. All data are presented as mean ± standard deviation (SD) based on three independent biological replicates. Figures were plotted in GraphPad Prism v. 8.0.1 (GraphPad Software, La Jolla, CA, USA) and TBtool (Chen et al., 2020a).

## Results

### Changes in Light Intensity, Temperature, and Humidity

**Figure 1:**
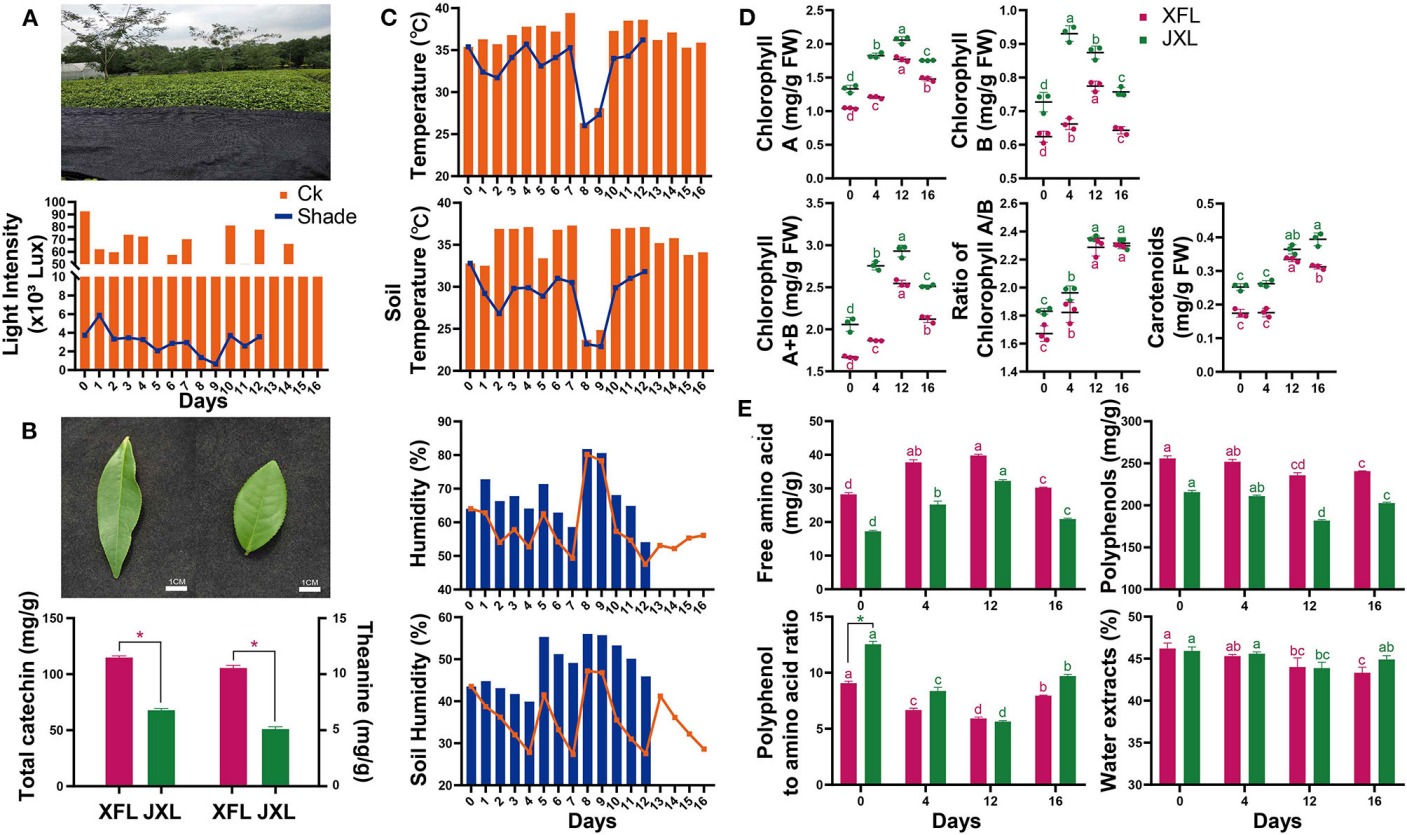
Shading photograph, light intensity **(A)**, leaf shape, the total catechin and theanine content of two tea cultivars **(B)**, air temperature and humidity, and soil temperature and humidity **(C)** were measured of tea plants. The contents of chlorophyll A, chlorophyll B, chlorophyll A/B, total chlorophyll, carotenoids **(D)** and free amino acids, tea polyphenols, polyphenol:amino acid ratio, and water extract **(E)** in the leaves of “Xiangfeicui” and “Jinxuan” (mean ± SD, three biological replicates per treatment groups) under shading treatments. Data were assessed by one-way ANOVA followed by Duncan's multiple range test. *p < 0.05. Ck, control check; Shade, shading treatment; XFL, leaf of “Xiangfeicui” cultivar; JXL, leaf of “Jinxuan” cultivar. Different letters in same color represent *p* < 0.05.

As shown in Figure 1A, the light radiation levels at canopy level for the unshaded controls and shaded plants were 13,040–92,530 lx and 650–5,870 lx, respectively. Thus, 95.12% of solar irradiation was blocked by shading. The average canopy air temperature and soil temperature under shading were 2.82 and 5.19°C lower than that in the control conditions, respectively. The average canopy air humidity and soil humidity under shading were 9.14 and 12.77% higher than that in the control conditions, respectively (Figure 1C). These indicate that shading significantly improves the growth environment of tea plants in summer and autumn.

### Changes in Photosynthetic Pigments and Ultrastructures

As shown in Figure 1D, the chlorophyll A and B levels were higher in JX leaves (JXL) than in XFC leaves (XFL). For both cultivars, the leaf chlorophyll A and B contents significantly increased under shading (*p* < 0.05). The total chlorophyll content and the chlorophyll A/B ratios increased with shading duration (*p* < 0.05). In both XFL and JXL, the carotenoid content did not changed but reached its maximum by the 12th day of shading (*p* < 0.05). After 4 days of light recovery, the chlorophyll A and B and total chlorophyll content decreased in both tea varieties (*p* < 0.05). These indicate that shading significantly enhances the degree of green in tea leaves.

**Figure 2:**
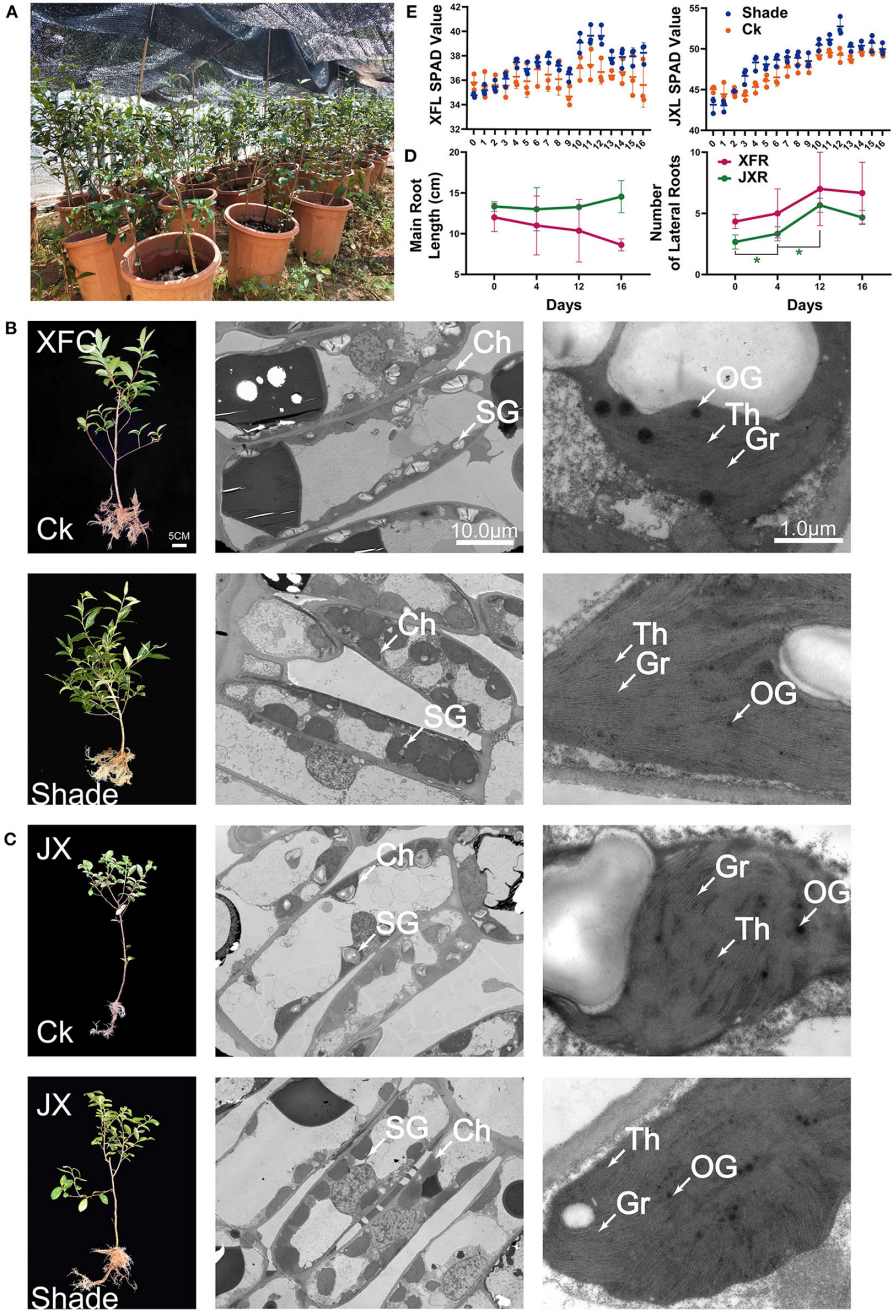
Schematic of pot experiment **(A)**. Whole plant phenotype; effect of shading on the ultrastructure of cell organelles in leaves of two tea cultivars **(B,C)**. The values of main root length, lateral root number **(D)**, and SPAD **(E)** of two tea cultivars (means ± SD, three biological replicates per treatment group) under shaded and unshaded conditions. Data were assessed by one-way ANOVA followed by Duncan's multiple range test. *p < 0.05. Ck, control check; Shade, shading treatment; XFC, “Xiangfeicui” cultivar; JX, “Jinxuan” cultivar; Ch, chloroplast; SG, starch granule; OG, osmiophilic granule; Gr, grana; Th, thylakoid; SPAD, soil and plant analyzer development, measuring relative chlorophyll content; XFL, leaf of “Xiangfeicui” cultivar; JXL, leaf of “Jinxuan” cultivar; XFR, root of “Xiangfeicui” cultivar; JXR, root of “Jinxuan” cultivar.

In pot experiment (Figure 2A), XFC and JX did not differ markedly in terms of their main root lengths (Figure 2E). However, the numbers of lateral roots in JX had significantly increased during shading (*p* < 0.05). In addition, we measured the SPAD value in XFL and JXL, and the trend was consistent with previous chlorophyll contents (Figure 2D). TEM was used to assess the effects of shading on leaf chloroplast ultrastructure (Figures 2B,C). Starch granules (SG), osmiophilic granules (OG), thylakoids (Th), and tightly stacked grana (Gr) were observed in both XFC and JX leaf chloroplasts. Grana stacking and thylakoids were enriched in both XFL and JXL during shading. The volume of chloroplasts increased remarkably, and the structure became more compact. These findings were consistent with the measured changes in chlorophyll content. However, the number of SG decreased after 4 days of shading and OG quantity and volume declined with shading duration.

### Changes in Polyphenols, Amino Acids, and Sensory Quality

As shown in Figure 1B, the total catechin and theanine contents in XFL were 1.69 and 2.07 times higher than in JXL (*p* < 0.05). At the same time, the polyphenol/amino acid ratios of XFL and JXL were 9.07 ± 0.18 and 12.54 ± 0.26 in the 0th day of shading, respectively (Figure 1E). The free amino acid content significantly increased while the polyphenol content significantly decreased after 12-day shading (*p* < 0.05). In both varieties, the polyphenol/amino acid ratios significantly decreased during shading (*p* < 0.05), and the lowest ratios of XFL and JXL were 5.92 ± 0.12 and 5.64 ± 0.09, respectively. After light recovery, the polyphenol, free amino acid content, and the polyphenol/amino acid ratio significantly increased to control levels (*p* < 0.05). The polyphenol/amino acid ratio is mainly responsible for umami taste and overall quality of the tea. Compared with XFL, JXL contain lower catechin and theanine contents, and higher polyphenol/amino acid ratio. However, shading promotes tea quality and affects the two varieties to different degrees. This is contrary to our results and needs to be further explored.

Shading treatments had a greater impact on the appearance, aroma, taste, and infused leaf quality of tea, but with less impact on the infusion color (Supplementary Table 3). Compared with unshaded tea, the leaves after 12 days of shading (12XFL and 12JXL) got the highest score: 90.7 ± 0.2 and 92.4 ± 0.2, respectively (*p* < 0.05). They had a strong aroma as well as a fresh and umami taste, in contrast to the control's slight aroma and pure and slightly astringent taste. These indicate that shading can significantly reduce the bitterness and astringency of tea, thus improving the taste and quality of summer and autumn green tea.

### Changes in Catechins, Caffeine, and Theanine Contents

To explore the differences in the biochemical components of unshaded and shaded leaves, the contents of catechins (C, EC, ECG, GCG, EGC, and EGCG), caffeine (CAF) and theanine in the tested tea leaf samples were determined. Table 1 shows that the concentration of theanine and caffeine in XFL and JXL increased significantly after 4- and 12-day shading (*p* < 0.05). Catechin contents in XFL and JXL at the 4th and 12th day were lower than in the unshaded leaves, while C, EC, ECG, EGCG content in two varieties had no significant change until the 12th day. Among them, non-galloylated catechins (C, EC, and EGC) contents in unshaded XFL and JXL were 1.58-, 1.71-, 3.82-, 1.33-, 1.49-, and 1.58-fold higher than in shaded leaves, respectively (*p* < 0.05, each). After light recovery, the contents of eight biochemical components tended to the original level (*p* < 0.05), while GCG showed a opposite trend. Catechins are mainly responsible for the bitter and astringent taste of the tea. Compared with unshaded tea, shaded tea of XFL and JXL usually contain lower levels of catechins, and provide a fresh flavor.

**Table 1:** Biochemical compositions of leaves in two tea cultivars under different shading periods.

**Cultivar**	**Shading periods (day)**	**Theanine**	**Caffeine**	**DL-C**	**EC**	**ECG**	**GCG**	**EGC**	**EGCG**
XFL	0	10.55 ± 0.23^bc^	35.58 ± 1.92^c^	6.48 ± 0.38^a^	7.32 ± 0.03^a^	11.21 ± 0.21^b^	6.82 ± 0.65^b^	32.52 ± 0.72^a^	50.50 ± 1.04^b^
	4	11.89 ± 0.09^a^	42.57 ± 0.47^b^	6.80 ± 0.06^a^	7.72 ± 0.47^a^	11.82 ± 0.75^b^	9.17 ± 0.96^a^	24.87 ± 0.64^b^	49.74 ± 0.31^b^
	12	10.90 ± 0.11^b^	51.83 ± 1.79^a^	4.09 ± 0.96^b^	4.29 ± 0.13^c^	9.16 ± 0.73^c^	5.38 ± 0.53^b^	8.51 ± 1.02^d^	31.36 ± 0.84^c^
	16	10.08 ± 0.48^c^	44.55 ± 0.99^b^	5.87 ± 0.28^a^	6.36 ± 0.44^b^	14.45 ± 0.26^a^	5.49 ± 0.82^b^	23.51 ± 0.33^c^	59.49 ± 1.17^a^
JXL	0	5.10 ± 0.20^b^	27.29 ± 1.29^c^	3.90 ± 0.57^a^	6.49 ± 0.47^a^	6.47 ± 0.37^bc^	3.57 ± 0.25^a^	19.51 ± 0.64^a^	27.86 ± 0.12^b^
	4	7.28 ± 0.23^a^	30.61 ± 1.64^b^	4.16 ± 0.31^a^	6.01 ± 0.68^a^	7.54 ± 1.10^b^	3.02 ± 0.09^b^	13.00 ± 0.33^c^	27.28 ± 0.54^b^
	12	7.74 ± 0.01^a^	34.73 ± 1.25^a^	2.93 ± 0.30^b^	4.35 ± 0.32^b^	5.90 ± 0.23^c^	2.90 ± 0.31^bc^	12.35 ± 0.22^c^	22.87 ± 1.20^c^
	16	5.07 ± 0.05^b^	31.75 ± 0.29^b^	4.35 ± 0.56^a^	6.16 ± 0.54^a^	11.75 ± 0.32^a^	2.58 ± 0.19^c^	16.05 ± 0.14^b^	39.26 ± 2.38^a^

*Concentrations (dry weight, mg/g). Data were presented as mean ± SD (standard deviation) and were assessed by one-way ANOVA followed by Duncan's multiple range test. DL-C, Catechin; EC, (–)-Epicatechin; ECG, (–)-Epicatechin gallate; GCG, (–)-Gallocatechin gallate; EGC, (–)-Epigallocatechin; EGCG, (–)-Epigallocatechin gallate; XFL, leaf of “Xiangfeicui”; JXL, leaf of “Jinxuan”*.

a,b,c *Different letters of same cultivar in row represent p < 0.05*.

### Metabolomics Analysis

#### Identification of Metabolites

**Figure 3:**
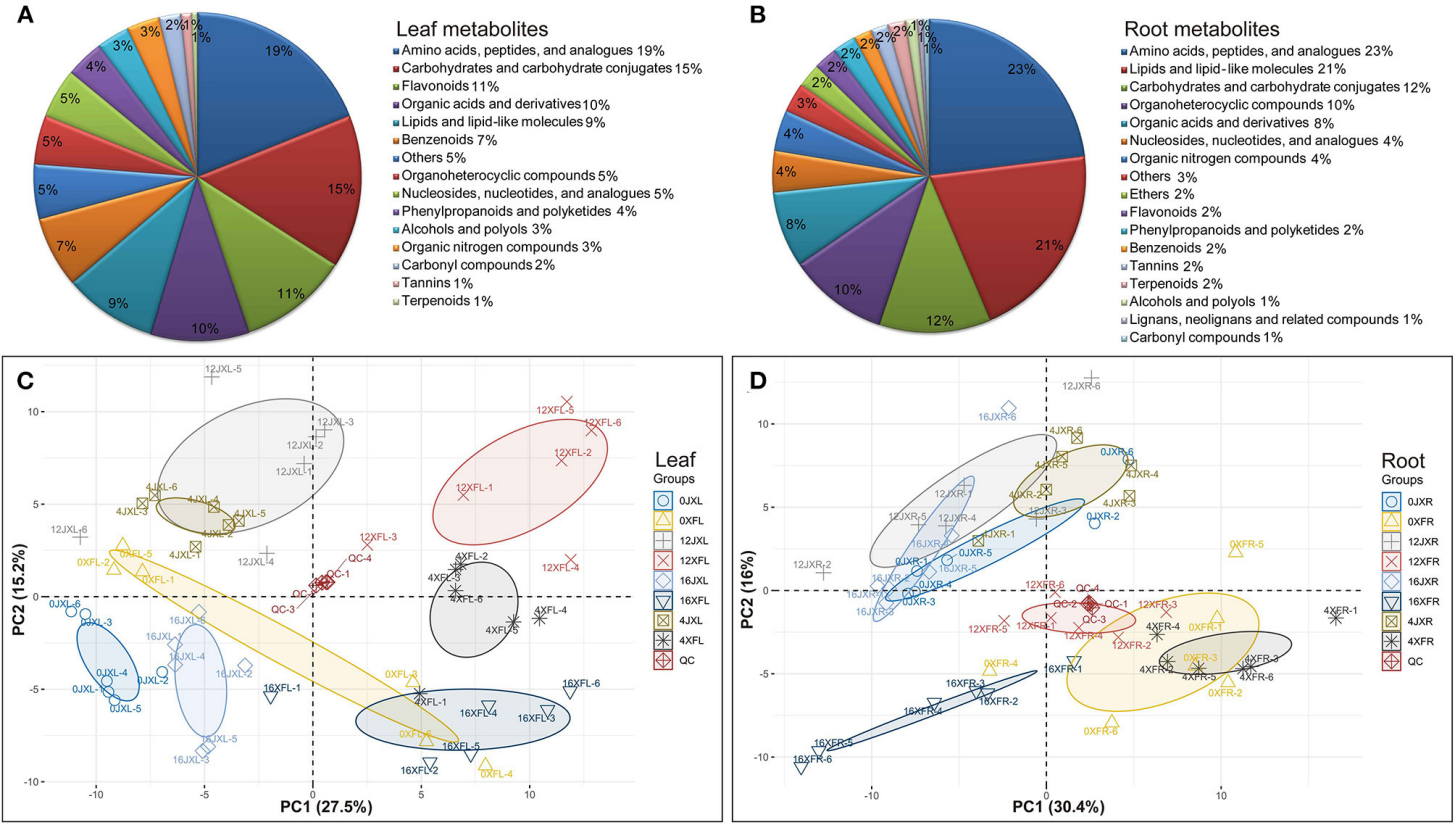
The classification of 185 differential metabolites in leaves **(A)** and 165 differential metabolites in roots **(B)**. PCA score plot of tea samples based on the relative variation of the leaf metabolites **(C)** and root metabolites **(D)** detected by UHPLC-Q TOF MS using the leaves and roots harvested from two cultivars under shading. The legend (Group) describes the abbreviations for the unshaded “Xiangfeicui” leaves (0XFL), XFC leaves after 4 days of shading (4XFL), XFC leaves after 12 days of shading (12XFL), XFC leaves after 4 days of regaining light (16XFL), unshaded “Jinxuan” leaves (0JXL), JX leaves after 4 days of shading (4JXL), JX leaves after 12 days of shading (12JXL), JX leaves after 4 days of regaining light (16JXL); XFC roots when the plants were unshaded (0XFR), XFC roots after 4 days of shading (4XFR), XFC roots after 12 days of shading (12XFR), XFC roots after 4 days of regaining light (16XFR), JX roots when the plants were unshaded (0JXR), JX roots after 4 days of shading (4JXR), JX roots after 12 days of shading (12JXR), and JX roots after 4 days of regaining light (16JXR). Each sample has six biological replicates (1–6). QC represents quality control.

In total, 185 differential metabolites in leaves and 165 differential metabolites in roots, were obtained for subsequent analysis (Supplementary Table 4). As shown in Figures 3A,B, leaf metabolites were divided into 15 categories, mainly including “carbohydrates and carbohydrate conjugates,” “amino acids, peptides, and analogs,” “nucleosides, nucleotides, and analogs,” “lipids and lipid-like molecules,” “organic acids and derivatives,” “benzenoids,” “organoheterocyclic compounds,” and “flavonoids”; root metabolites were divided into 17 categories, mainly including “amino acids, peptides, and analogs,” “carbohydrates and carbohydrate conjugates,” “lipids and lipid-like molecules,” “organonitrogen compounds,” “nucleosides, nucleotides, and analogs,” and “organic acids and derivatives.”

#### Multivariate Analysis of Extracts From the Leaves and Roots

PCA and OPLS-DA were performed to visualize general clustering patterns and reveal differences between tea cultivars in terms of their leaf and root metabolomes over various shading durations. The PCA model was based on the secondary metabolites detected by UHPLC-Q-TOF MS and showed a separation trend in the leaves and roots of the tea cultivars. Figures 3C,D shows that XFL and XFC roots (XFR) were separated to the right side of the PC1 axis while JXL and JX roots (JXR) were separated to the left side of the PC1 axis. Thus, PC1 was separated by tea variety and accounted for 27.5 and 30.4% of the difference, respectively. Moreover, the samples classified by shading treatment were separated in the PC2 axis and 12JXL and 12XFL were further from their unshaded controls than 4JXL and 4XFL were. The light recovery samples (16JXL and 16XFL) were relatively closer to the unshaded controls (0JXL and 0XFL). Thus, PC2 was separated by shading duration and accounted for 15.2 and 16% of the difference, respectively. Multivariate analysis indicate that shading modulates metabolite levels in tea leaves and roots. Therefore, we conducted a further analysis of the metabolomics around metabolite pathways.

#### Analysis of Differentially Expressed Metabolites (DEMs)

**Figure 4:**
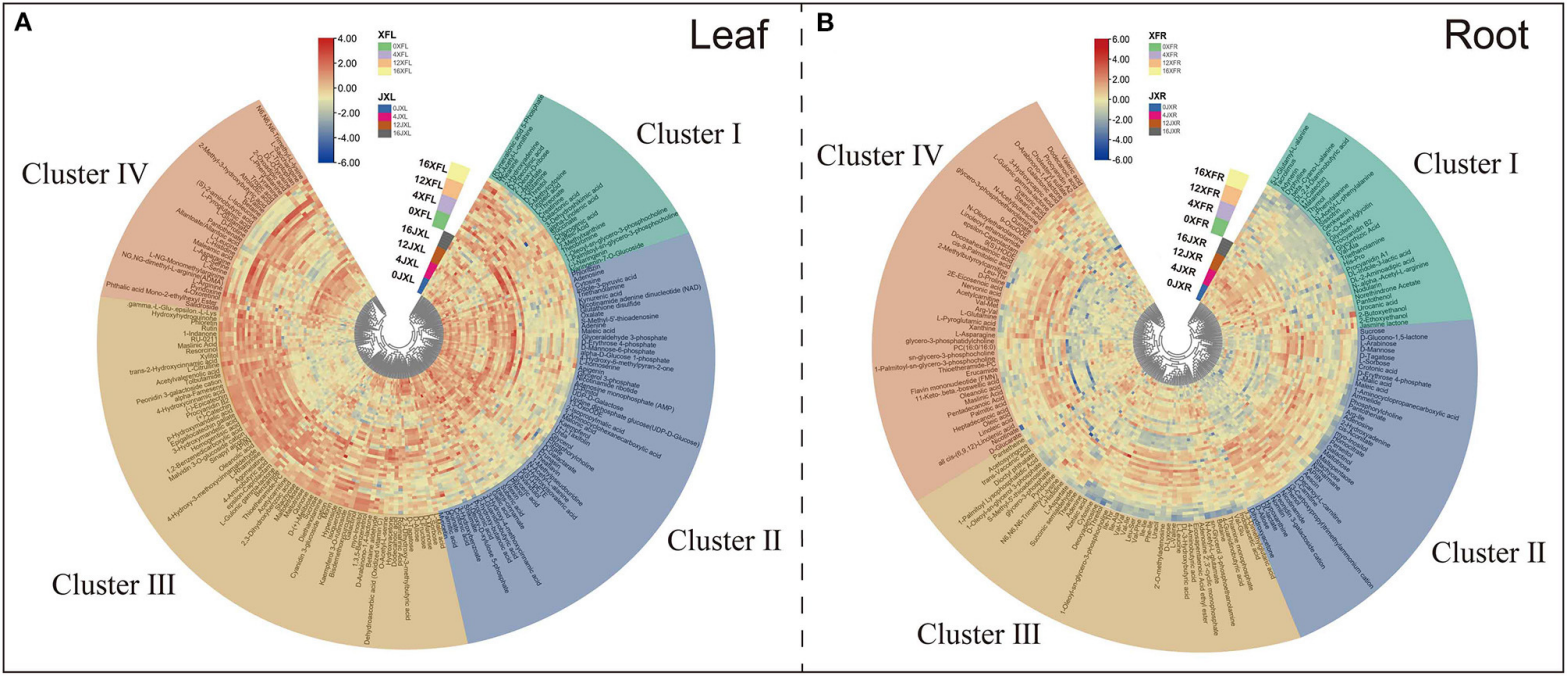
Hierarchical clustering of 185 leaf differential metabolites **(A)** and 165 differential root metabolites **(B)** identified from two tea cultivars with different shading periods. From center to edge, unshaded “Jinxuan” leaves (0JXL), JX leaves after 4 days of shading (4JXL), JX leaves after 12 days of shading (12JXL), JX leaves after 4 days of regaining light (16JXL), the unshaded “Xiangfeicui” leaves (0XFL), XFC leaves after 4 days of shading (4XFL), XFC leaves after 12 days of shading (12XFL), XFC leaves after 4 days of regaining light (16XFL); JX roots when unshaded (0JXR), JX roots after 4 days of shading (4JXR), JX roots after 12 days of shading (12JXR), and JX roots after 4 days of regaining light (16JXR), XFC roots when unshaded (0XFR), XFC roots after 4 days of shading (4XFR), XFC roots after 12 days of shading (12XFR), XFC roots after 4 days of regaining light (16XFR). Each sample has six biological replicates (1–6). The metabolite levels changed, and they could be organized into four clusters named clusters I, II, III, and IV. Blue indicates relatively low intensity, while red indicates relatively high intensity.

To study the effects of shading on tea cultivar leaf and root metabolic profiling, we selected various metabolites based on VIP >1 and Fold change ≥1.5 or ≤ 0.67 between samples (Supplementary Table 4). We divided the screening samples into two parts: one is the comparison between XFC and JX, and the other is the comparison of different shade stages. In total, 111 leaf DEMs and 112 root DEMs between varieties, and 173 leaf DEMs and 144 root DEMs with different shade stage were identified, respectively. As shown in Figures 4A,B, we organized and visualized all DEMs through hierarchical clustering analysis (HCA) using the Euclidean distance coefficient and the complete linkage method. Based on the relative differences in metabolites between cultivars and among shading treatments, the compounds in the leaves and roots were grouped into four clusters (I, II, III, and IV). In Figure 4A, cluster I metabolites under shading (days 4 and 12) were more concentrated than those under light exposure, and the metabolite levels in XFL were higher than in JXL, such as Theanine, L-Aspartate, and L-Glutamine. Cluster II, included Kaempferol, Theaflavin, and Gallic acid, occurred at higher levels in the JXL than in the XFL, and the metabolites in 0XFL were the highest level of XFL. Cluster III comprised catechins, α-Farnesene, 4-Aminobutyric acid, and Sucrose, were detected at higher levels in the XFL than in the JXL in all stages, and decreased their contents during the shading period. In cluster IV, the metabolite levels significantly increased under shading and reached the highest expression at the 12th day (12XFL and 12JXL), such as L-Asparagine, L-Glutamate, and L-Phenylalanine; these metabolites had higher level in XFL than in JXL.

Figure 4B shows that cluster I included (+)-Catechin, L-Phenylalanine and Jasmine lactone, and they occurred at higher levels in JXR than in XFR. Cluster II, included Sucrose, D-Mannose, and Myo-inositol, occurred at higher levels in XFR than in JXR, and the metabolite levels were decreased after the 12-day shading. Cluster III comprised Betaine, 4-Aminobutyric acid, Theanine, and L-Aspartate, were detected at higher levels in XFR than in JXR in the 0th and 4th day, and reached their highest contents after the 4-day shading. In cluster IV, the metabolites were detected at higher levels in 4XFR than in 4JXR, such Linoleic acid, Oleic acid, Oleanolic acid, L-Asparagine, and L-Glutamine. These results showed the changes of metabolites in tea leaves and roots to possibly be due to the difference in variety and shading duration.

#### Venn Analysis of DEMs in Leaves and Roots

##### DEMs Exposed to Shading at Various Durations

**Figure 5:**
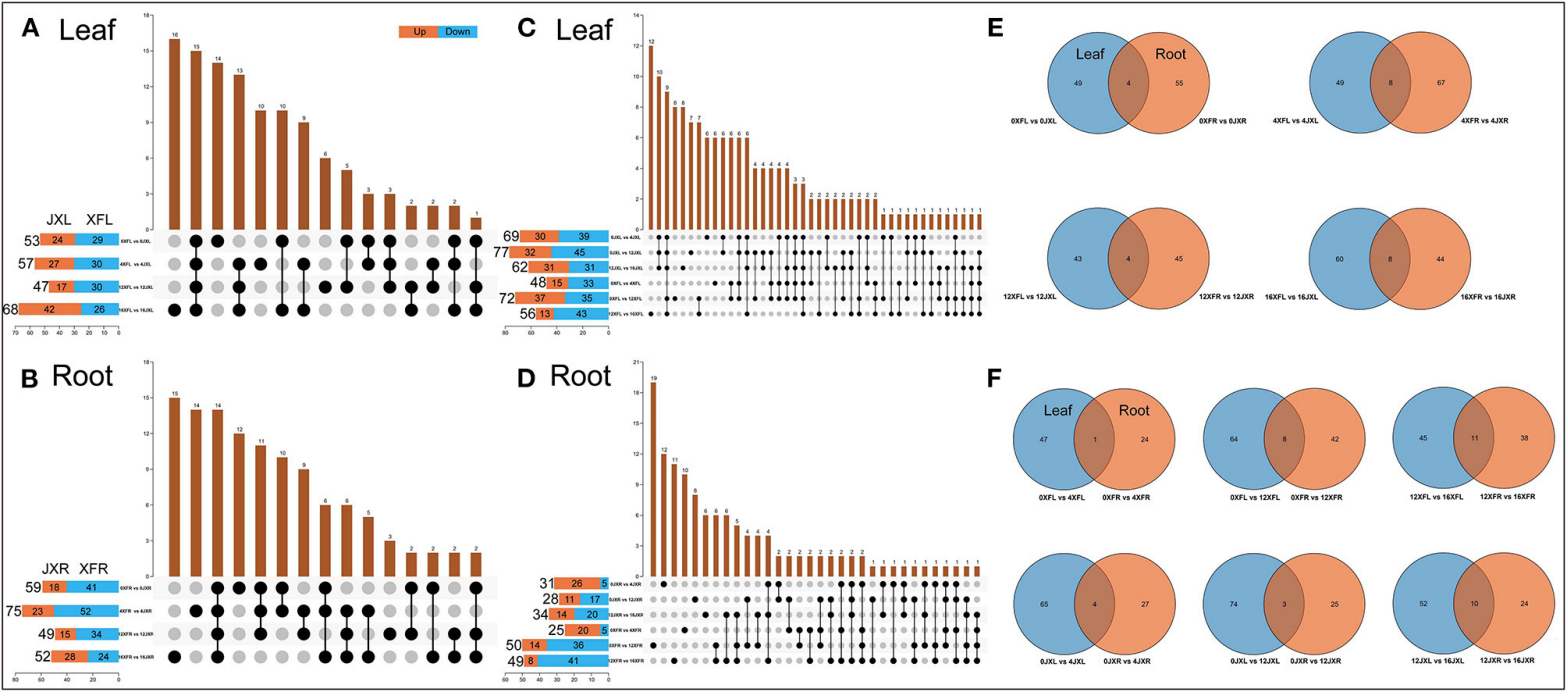
The Venn analysis of differential metabolites involved in leaves and roots of two tea cultivars. The value of the row represents the total number of differential metabolites and “vs.” means that the latter value to the previous value. Orange means the latter value is larger, while blue means the previous is larger. The value of the columns indicates the number of metabolites that are common to several sets of comparisons. **(A)** Comparison of leaf differential metabolites among cultivars in different shading periods. **(B)** Comparison of root differential metabolites among cultivars in different shading periods. **(C)** Comparison of leaf differential metabolites between different shading periods within the same cultivar. **(D)** Comparison of root differential metabolites between different shading periods within the same cultivar. **(E)** Differential metabolites shared in leaves and roots among two cultivars during the same shading period. **(F)** Differential metabolites shared in leaves and roots with the different shading duration. XFL, the leaf of “Xiangfeicui” cultivar; JXL, the leaf of “Jinxuan” cultivar; XFR, the root of “Xiangfeicui” cultivar; JXR, the root of “Jinxuan” cultivar.

We conducted upset plot analysis of DEMs involved in leaves and roots. As shown in Figure 5A, the light recovery stage in leaves (16XFL vs. 16JXL) had the highest number of total DEMs and unique DEMs (68 and 16, respectively). There were 15 common DEMs to all stages in leaves, such as (+)-Catechin, (–)-Epicatechin, Epigallocatechin gallate, and Procyanidin B2. A total of 27 DEMs were caused by shading and relight treatments (14 unique DEMs in 0XFL vs. 0JXL and 13 common in other three stages), including L-Arginine, Sucrose, Kaempferol, Rutin, Urea, and Theaflavin. Sixteen unique DEMs under shading (day 4 and 12) included L-Leucine, D-Proline, and L-Asparagine, and 10 unique DEMs with non-shaded treatment (day 0 and 16) included Theanine, Indole-3-pyruvic acid, and D-Mannose. As shown in Figure 5B, the number of up-regulated DEMs were more in XFR than in JXR at all stages except the 16th day. There were 26 DEMs (15 unique DEMs in 16XFR vs. 16JXR and 11 common in other three stages) in roots during the light recovery stage, such as myo-Inositol, Oleic acid, Stachyose, L-Pyroglutamic acid, and D-Proline. Fourteen DEMs included Linoleoyl ethanolamide, Linoleic acid, and Nicotinamide, were present at all stages. The shading and relight treatment caused 18 unique DEMs, including D-Allose, Indoleacetic acid, 4-Aminobutyric acid, and Procyanidin B2. A total of 26 DEMs were caused by shading (day 4 and 12), such as four dipeptides (Leu-Thr, Arg-Val, Arg-Ile, Leu-Ser), Theanine, DL-Indole-3-lactic acid, and L-Glutamine.

As shown in Figure 5C, the amount of leaf DEMs in two varieties with different shading durations both showed a trend of first increase (day 4 to day 12) and then decrease (light recovery). The 12-day shading treatment promoted the more up-regulated DEMs in 0XFL vs. 12XFL. There were 12 unigue DEMs in 12XFL vs. 16XFL, such as DL-Serine, L-Glutamine and 4-Aminobutyric acid. Ten common DEMs included Sucrose, Isoquercitin, D-Tagatose, Kaempferol, and Indole-3-pyruvic acid, were caused by shading and relight treatment in JXL. Twelve common DEMs were present at all durations in XFL and JXL, including Tyramine, L-Leucine, L-Phenylalanine, Betaine, L-Histidine, L-Isoleucine, and D-Fructose. The 12-day shading treatment caused 8 and 7 unigue DEMs in XFL and JXL, respectively; the 4-day shading treatment caused both 4 unigue DEMs in XFL and JXL. There were both 6 common DEMs at the 4th and 12th day in XFL [such as Theaflavin, (–)-Epicatechin and 7-Methylxanthine] and JXL (such as Kaempferol 3-O-rutinoside and Apigenin). As shown in Figure 5D, the amount of down-regulated DEMs in XFR and JXR increased after the 12-day shading and relight treatment. The 12-day shading caused the highest amount of unigue DEMs in 0XFR vs. 12XFR, including Stearic acid, D-Mannose, L-Asparagine, Indoleacetic acid, Adenosine, and D-Tagatose; 8 unigue DEMs in JXR, such as Theanine. There were 12 unigue DEMs in 0JXR vs. 4JXR at the 4th day of shading, such as 4-Guanidinobutyric acid, N-Acetyl-L-glutamate, and Procyanidin B2; 10 unigue DEMs in 0XFR vs. 4XFR, including Val-Met, Nicotinate, and DL-Indole-3-lactic acid. A total 6 common DEMs were present during the relight stage between XFR and JXR, such as L-Leucine, D-Proline, and L-Aspartate. These results indicated that changes of tea plant metabolites under shading were remarkably associated with cultivar and shade duration.

##### Common DEMs in Leaves and Roots

As shown in Figure 5E, we obtained the common DEMs in leaves and roots by comparing the two varieties at various shading durations. In the 0th day of shading, D-Allose, D-Tagatose, and (+)-Catechin were the common DEMs. There were 8 common DEMs in the 4th day, including Sucrose, Stachyose, Procyanidin B2, (+)-Catechin, and D-Proline. In addition, Procyanidin B2 was present at all stages except the 0th day, and expressed higher level in XFL than in JXL but lower level in XFR than in JXR. As shown in Figure 5F, (+)-Catechin was relatively more abundant both in two varieties at the 4th day. Betaine was relatively more abundant in JXL and JXR after 4- and 12-shading treatments, and 8 common DEMs included L-Asparagine, L-Histidine, and L-Sorbose in XFL and XFR under 12-day shading. The light recovery stage induced the most common DEMs in XFL-XFR and JXL-JXR (11 and 10, respectively), such as L-Leucine, L-Aspartate, and D-Proline. These results showed the shading mediated the joint metabolic regulation of tea leaves and roots.

### Gene Expression of Main Tea Quality-Metabolites

**Figure 6:**
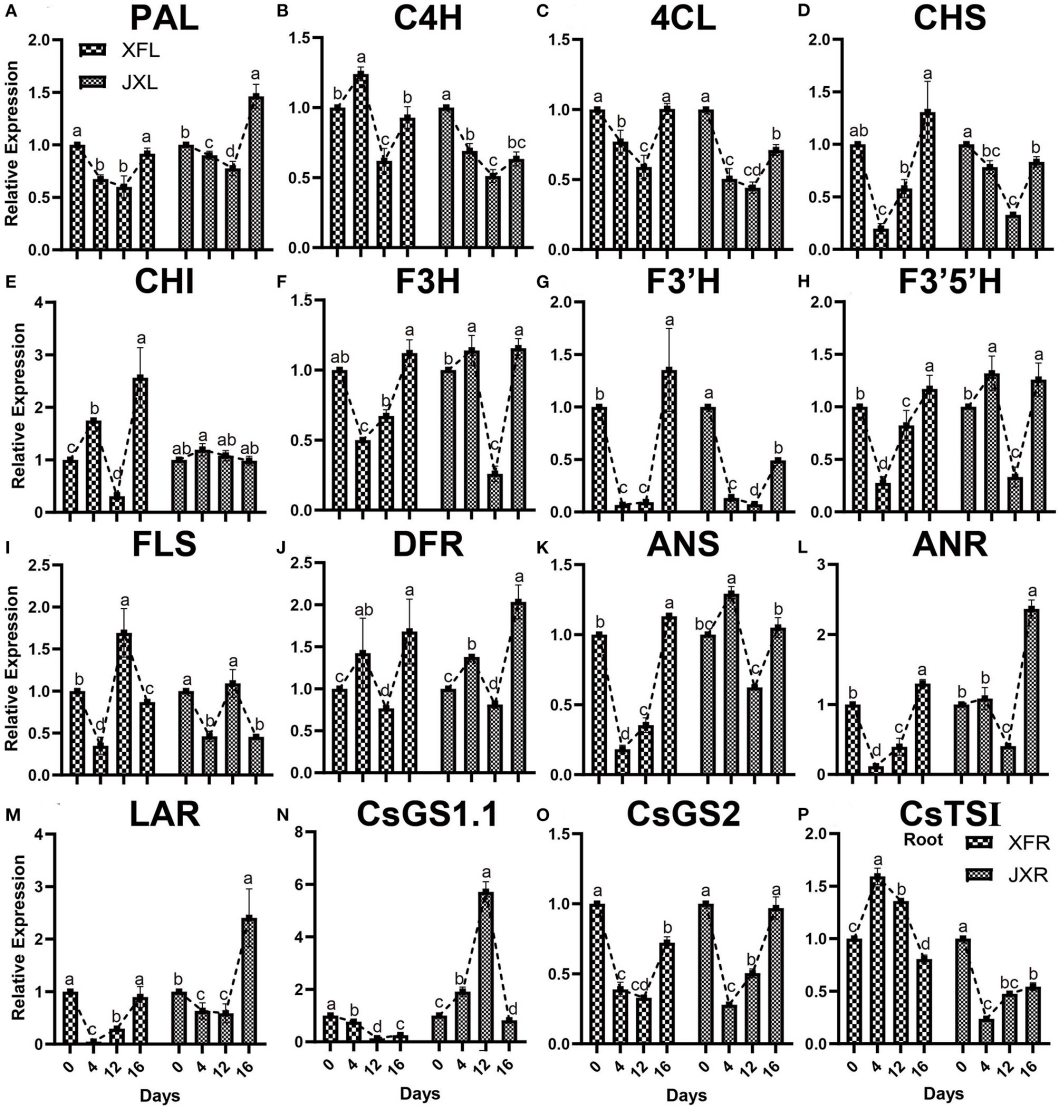
Expression of a selected set of genes in the leaves and roots of two cultivars (“Xiangfeicui” and “Jinxuan”) exposed to different shading treatments. The expression of each gene was calculated relative to GAPDH gene. **(A)** *PAL*, phenylalanine ammonia lyase; **(B)** *C4H*, cinnamate 4-hydroxylase; **(C)** *4CL*, 4-coumarate–CoA ligase; **(D)** *CHS*, chalcone synthase; **(E)** *CHI*, chalcone isomerase; **(F)** *F3H*, flavanone 3-hydroxylase; **(G)** *F3*′*H*, flavonoid 3′-monooxygenase; **(H)** *F3*′*5*′*H*, flavonoid 3′,5′-hydroxylase; **(I)** *FLS*, flavonol synthase; **(J)** *DFR*, dihydroflavonol-4-reductase; **(K)** *ANS*, anthocyanidin synthase; **(L)** *ANR*, anthocyanidin reductase; **(M)** *LAR*, leucoanthocyanidin reductase; **(N)** *CsGS1.1*, glutamine synthetase 1.1; **(O)** *CsGS2*, glutamine synthetase 2; **(P)** *CsTSI*, theanine synthetase I (in root). Data were assessed by one-way ANOVA followed by Duncan's multiple range test. XFL, the leaf of “Xiangfeicui” cultivar; JXL, the leaf of “Jinxuan” cultivar; XFR, the root of “Xiangfeicui” cultivar; JXR, the root of “Jinxuan” cultivar.

qRT-PCR was used for verification of the key genes, which regulated important tea quality-metabolites, including 13 genes related to flavonoid biosynthesis and 3 amino acid biosynthesis related genes. As presented in Figures 6A–M, all genes except FLS involved in flavonoid metabolism in XFL and JXL, including phenylalanine ammonia lyase (*PAL*), 4-coumarate: CoA ligase (*4CL*) gene, chalcone synthase (*CHS*) gene, flavonoid 3′-monooxygenase (*F3*′*H*) gene and leucoanthocyanidin reductase (*LAR*) gene, were downregulated under 12-day shading. The shading treatment downregulated flavonoid related genes, indicating that the significant decrease in catechin contents was mainly attributed to the reduction in bitterness of the tea. In addition, the biosynthetic genes, including flavanone 3-hydroxylase (*F3H*) gene, flavonoid 3′,5′-hydroxylase (*F3*′*5*′*H*) gene, dihydroflavonol-4-reductase (*DFR*) gene, anthocyanidin synthase (*ANS*) gene, and anthocyanidin reductase (*ANR)* gene, were upregulated in JXL, and cinnamate 4-hydroxylase (*C4H*) gene, chalcone isomerase (*CHI*) gene, and *DFR* were upregulated in XFL at the 4th day, which might be due to the stress response to shading of tea plants. As shown in Figures 6N–P, Glutamine synthetase 2 (*CsGS2*) gene that mediated the theanine biosynthesis in XFL and JXL were downregulated under shading, while glutamine synthetase 1.1 (*CsGS1.1*) expression increased remarkably in JXL under shading. Interestingly, our results showed that theanine synthetase I (*CsTSI*) gene was significantly up-regulated in XFR under shading. This suggested that shading could lead to increased expression of specific amino acid synthesis genes in tea leaves and roots but different in various cultivars. These results indicated that expression pattern of these genes was consistent with related metabolites.

## Discussion

### Shading Periods Dramatically Improved Green Tea Quality by Affecting the Photosynthesis, Biochemical Composition, and Gene Expression

Hot and dry weather tends to make green tea bitter and astringent (Li X. et al., 2016). However, the present study demonstrated that shading helped optimize the light intensity, temperature, and humidity of the environment in which the tea was grown. The level of chlorophyll and carotenoids, which are the major pigments that influence the tea leaf color, are participated in light harvesting and essential for photoprotection against excessive illumination (Chen et al., 2021). In tea plants, reduced light intensity caused by shading induces genes, transcription factors, and phytohormones involved in chlorophyll biosynthesis (Liu et al., 2020). In the present study, shading significantly increased the chlorophyll and carotenoid contents of both XFL and JXL in the field trial. However, the carotenoid content was only decreased in XFL after relight treatment, indicating that the light recovery treatment might differentially regulate the accumulation of carotenoid pigments in the JX cultivar, which consisted of low polyphenol and amino acid levels (Song et al., 2017). Simultaneously, TEM analysis showed that shading markedly increased the volume of chloroplasts in the mesophyll cells and made the grana and thylakoids compact, indicating that shading promoted chloroplast development and led to an increase in photosynthetic pigments (Chen et al., 2017; Gao et al., 2021).

The polyphenols and amino acids are important components of tea taste composition (Zhu et al., 2017). Our results showed that the polyphenol contents were significantly decreased in 12-day shaded XFL and JXL than in 4-day shaded leaves, and the amino acid contents increased with shading duration. Simultaneously, in the sensory evaluation, we observed that shaded green tea had a higher overall quality score than unshaded green tea and JX scored higher than XFC after 12 days of shading, indicating that the shading treatment might improve the tea quality and differentially regulate the content of carbon and nitrogen compounds for metabolic regulation (Li et al., 2020). The polyphenol/amino acid ratio is an important indicator to measure the quality of tea (Zhang Q. et al., 2021). XFC was a high polyphenol and amino acid contents variety with lower polyphenol/amino acid ratio, while JX was a low polyphenol and amino acid contents variety with higher polyphenol/amino acid ratio. In this study, the polyphenol/amino acid ratios were both decreased in XFL and JXL under shading, while the greater decline of the ratio was observed in 12-day JXL. Interestingly, JX is also suitable for black and oolong tea production. The results of the present study underscore the fact that high-quality green tea can be prepared from non-green tea varieties (Zhang G. et al., 2020). Altogether, these findings showed significant differences between the two cultivars, suggesting that the shading treatment might play important roles in JX cultivar because it had low polyphenol and amino acid contents (Li et al., 2020).

Shading improved the types and proportions of the secondary metabolites conducive to high tea quality, and promoted nitrogen metabolism in tea leaves possibly by increasing proteolysis (Yu and Yang, 2020). However, in this study, the levels of biosynthesized non-protein amino acids (Theanine) significantly increased in shaded XFL and JXL except the 12-day shaded XFL, indicating that long-time shading treatment might inhibit the increase of theanine level in XFL, which had high polyphenol and amino acid contents (Yang et al., 2021). In addition, it might have been an increase in protein hydrolysis that explained the measured amino acid distributions and levels (Chen et al., 2017). However, we found that *CsGS1.1* and *CsTSI* were upregulated in shaded JXL and XFR, respectively, which was consistent with previous results (Fu et al., 2021). Thus, shading may, in fact, have induced certain amino acid synthase genes in the leaves and roots (Liu et al., 2017). Caffeine contributes to the intensity of tea flavor, which increased under different shading periods. Extensive study has showed that high light intensity increases the expression of the structural genes and the activity of several important enzymes associated with the biosynthesis of flavonoids, which lead to high contents of catechins (Ye et al., 2021). Recently, some study found that shading tea plants remarkably decreased the catechin contents in tea buds (Zhang et al., 2022). Certain studies suggested that low light intensity downregulates the signal transduction pathways mediated by UVR8 and HY5 and suppresses transcription factors such as MYB (Liu et al., 2018; Zhao et al., 2021). However, we found that the 4-day shading treatment only lowered the EGC in XFL and JXL, while the 12-day shading treatment significantly decreased most catechin contents, including C, EC, ECG, GCG, EGC, and EGCG, indicating that shading treatment significantly reduced the bitterness and astringency by decreasing catechin contents and short-time shading might have slight effect on catechins of tea plants (Lin et al., 2021). In addition, our study measured the expression of 13 flavonoid biosynthesis genes, and they were differentially downregulated in the leaves of both cultivars under shading. However, some gene expression showed a significant upward trend in 4-day shaded leaves, including *C4H, CHI*, and *DFR* in XFL and *F3H, F3*′*5*′*H, DFR*, and *ANS* in JXL, respectively, which was consistent with the result that catechins only declined after 12-day shading. These results revealed that the observed decline in flavonoid content might be the result of regulatory gene downregulation under shading (Yu et al., 2021). Thus, the findings indicated that the control of the light intensity regulates the composition of tea quality-compounds in different tea plant varieties.

### The Differences in Metabolic Pathways of Two Tea Varieties Under Different Shading Periods

Shading affects both the aerial and belowground environments. However, researchers seldom link the metabolic changes that occur in leaves and roots with shading. In this study, we identified and measured carbon and nitrogen metabolites in both organs. Carbon-based compounds include soluble sugars and starch that serve as substrates for tea polyphenol biosynthesis via the shikimic acid and phenylpropane pathways (Li X. et al., 2019). Amino acids and caffeine are major nitrogen-based compounds; amino acids are synthesized via glycolysis, TCA cycle, and the oxidative pentose phosphate pathway, and caffeine is produced by purine and pentose phosphate pathway (Chen et al., 2020b; Yang et al., 2020). In addition, lipid metabolism associated with tea aroma, synthesized via glycolysis and amino acid pathways (Gai et al., 2020). Thus, the carbon and nitrogen metabolisms are closely integrated. To analyze the different metabolites between the two tea varieties, we proposed different metabolic pathways with reference to the KEGG database and other literatures.

#### Sugar Metabolism

**Figure 7:**
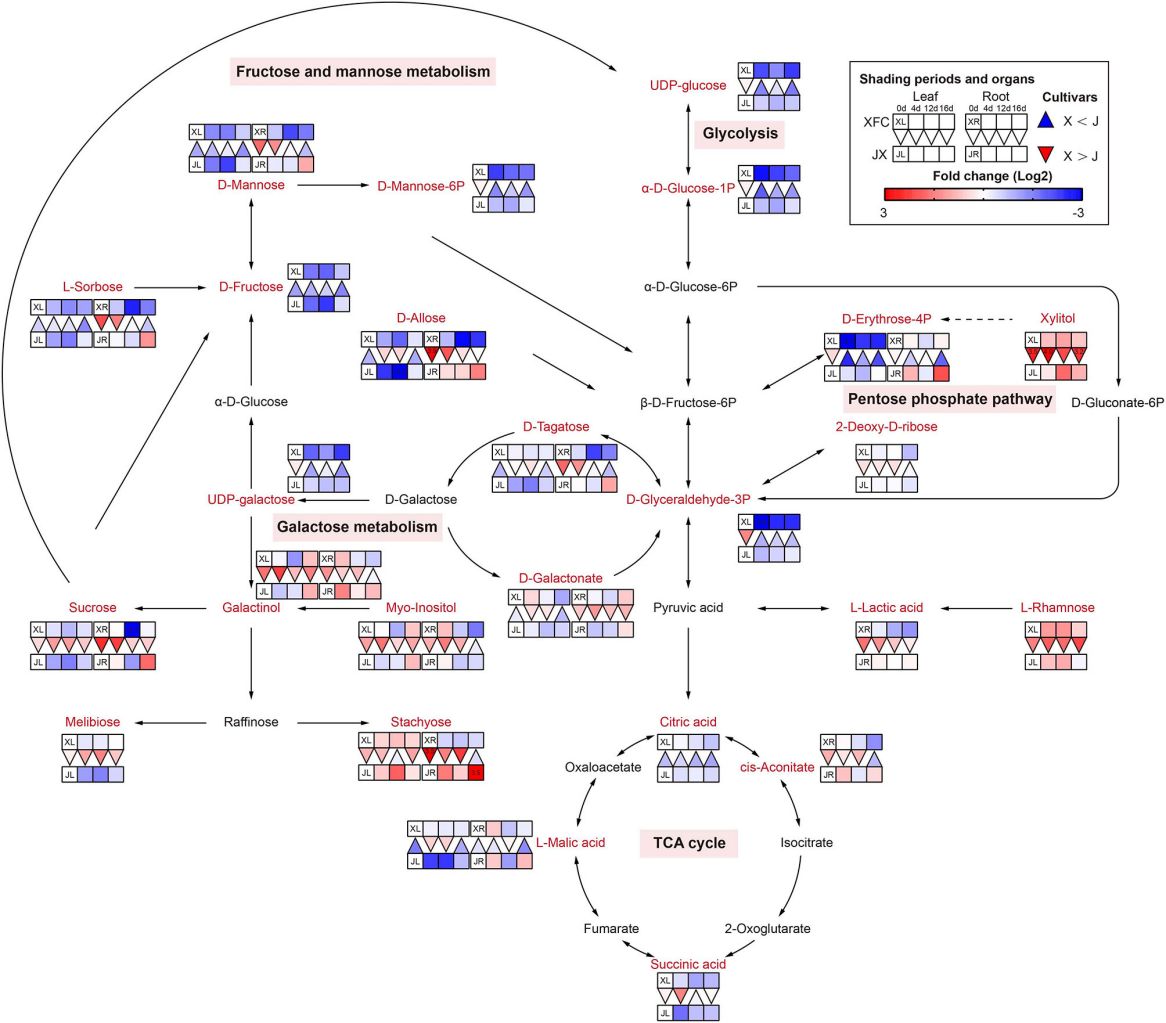
The metabolite differences during sugar metabolism in the two tea cultivars under different shading periods. The identified metabolites are marked with a red color. In the legend, the squares at two lines express the different cultivars, “Xiangfeicui” (XFC) and “Jinxuan” (JX). The squares at eight columns express the different shading periods. The squares in the left part represent the leaves and the right part represents the roots. Blue indicates relatively low and red indicates relatively high intensity. The red triangle indicates relatively higher intensity of metabolites in XFC (X) than in JX (J), and the inverted blue triangle indicates relatively lower intensity of metabolites in XFC (X) than in JX (J). The degree of change was described with the depth of color, and the depth of the colors is based on the log2-fold change value between the samples. XL, leaf of “Xiangfeicui”; XR, root of “Xiangfeicui”; JL, leaf of “Jinxuan”; JR, root of “Jinxuan”.

Sugars furnish metabolic energy for plants and serve as substrates and signaling molecules in various metabolic pathways, and could supply carbon skeletons for the synthesis of amino acids and nucleotides (Ruan, 2014). The glycolysis, tricarboxylic acid (TCA) cycle, galactose metabolism, pentose phosphate pathway, and fructose and mannose metabolism are the main pathways in the carbohydrate metabolism (Figure 7). Interestingly, we found that there were obvious differences of leaves and roots between XFC and JX under different shading periods. Glucose is an initial substrate for glycolysis, and other metabolites are known as intermediate product (Liu X. et al., 2016). We found that most metabolites of XFL in glycolysis and pentose phosphate pathway, including UDP-glucose, α-D-Glucose-1P, D-Glyceraldehyde-3P, and D-Erythrose-4P, had lower intensities under shading treatments. Concordantly, we found that the root metabolites in XFR such as Sucrose, L-Sorbose, D-Mannose, D-Tagatose, and D-Allose, showed decline to a greater extent than in the JXR under 12-day shading. In addition, the XFL had higher levels on intermediate production than the JXL, including Stachyose, L-Rhamnose, and Xylitol. Interestingly, we found significant increasing trends of JXR after relight treatment in sugar pathways and TCA cycle, such as Sucrose, Stachyose, D-Erythrose-4P, D-Allose, L-Sorbose, D-Mannose, L-Malic acid, and cis-Aconitate. Under natural conditions, the tea plants were shown to have higher contents of sugar, such as glucose and fructose derived from the hydrolysis of sucrose, which generally act as osmoprotectants and are involved in the glycolytic pathway (Dumont and Rivoal, 2019). To supplement their carbon skeletons during shading, the plants hydrolyzed large amounts of polysaccharides into soluble sugars and decreased their overall sucrose content (Du et al., 2020). Our results indicated that the shading treatment showed remarkably negative effect on sugar contents but varied significantly among the varieties. Under shade conditions, the need for energy by XFC is lower than JX and downstream products wound be enhanced, while JX were more sensitive to light recovery changes. The reduction in the synthesis of glucose might cause a feedback mechanism by shifting stored glucose to amino acid metabolism instead of normal carbon metabolism, which leads to the accumulation of amino acids (Li et al., 2020).

#### Lipid Metabolism

**Figure 8:**
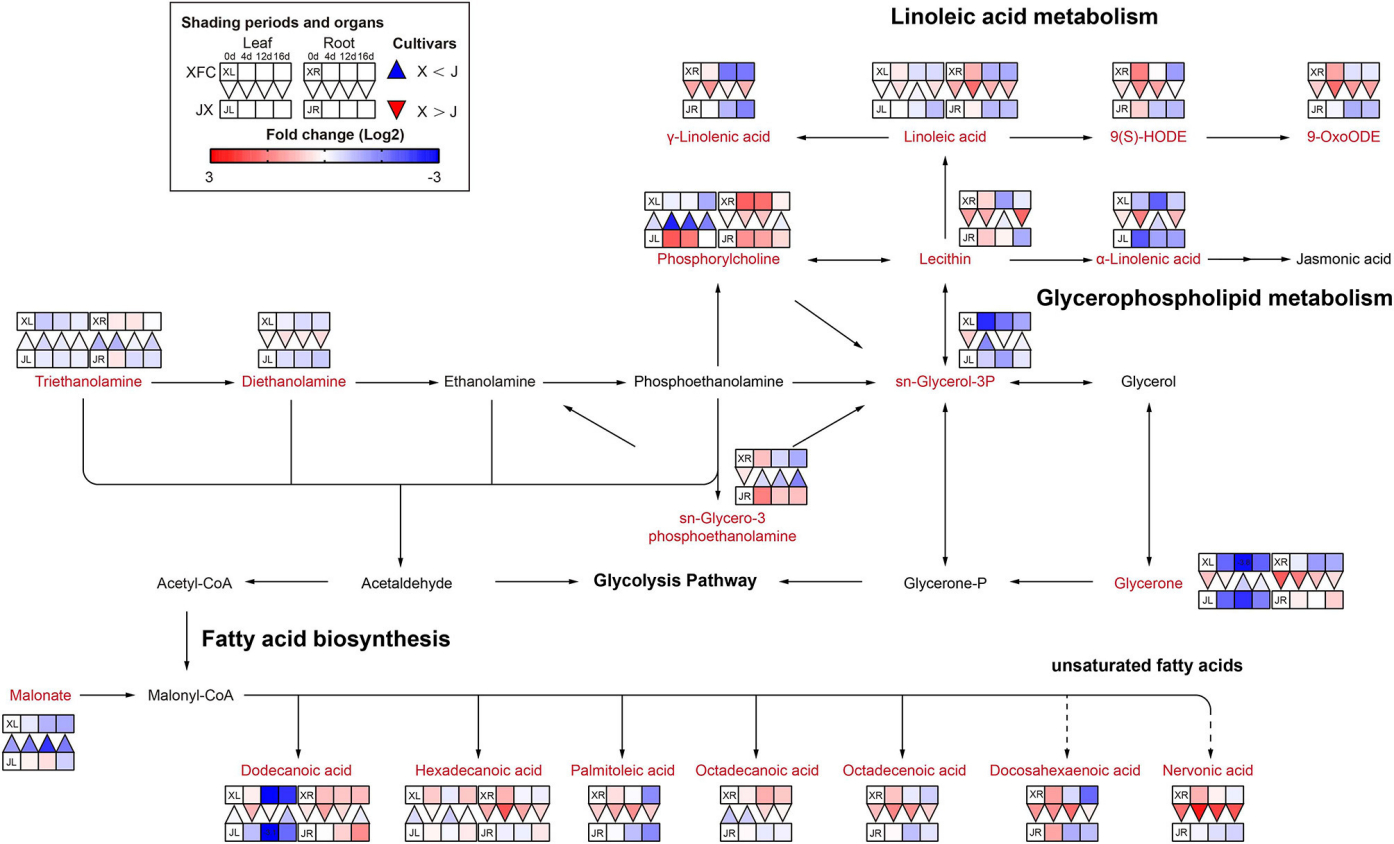
The metabolite differences during lipid metabolism in the two tea cultivars under different shading periods. The identified metabolites are marked with a red color. In the legend, the squares at two lines express the different cultivars, “Xiangfeicui” (XFC) and “Jinxuan” (JX). The squares at eight columns express the different shading periods. The squares in the left part represent the leaves and the right part represents the roots. Blue indicates relatively low and red indicates relatively high intensity. The red triangle indicates relatively higher intensity of metabolites in XFC (X) than in JX (J), and the inverted blue triangle indicates relatively lower intensity of metabolites in XFC (X) than in JX (J). The degree of change was described with the depth of color, and the depth of the colors is based on the log2-fold change value between the samples. XL, leaf of “Xiangfeicui”; XR, root of “Xiangfeicui”; JL, leaf of “Jinxuan”; JR, root of “Jinxuan”.

The lipids are the primary precursor of aromatic volatiles compounds and have an important cold-tolerance role in tea plants (Wang et al., 2020). They can generate other aroma compounds after oxidative degradation, among which α-linolenic acid, linoleic acid, oleic acid, and palmitic acid are the precursors of six to ten carbon aroma compounds (Liu M. Y. et al., 2017). Lipid metabolism is comprised of several pathways, including linoleic acid metabolism, glycerophospholipid metabolism and fatty acid biosynthesis (Figure 8). Most lipids only were detected in the root and had a higher levels after 4-day shading treatment in XFR than in JXR, including γ-Linolenic acid, Linoleic acid, 9(S)-HODE, 9-OxoODE and all fatty acids (except the Octadecanoic acid), and the same occurred in the leaves of XFC. However, the longer shading treatment could significantly reduce the lipid levels in the leaves and roots of XFC and JX. Linoleic acid is an important precursor of aroma compounds (Guo et al., 2020). In Figure 8, the Linoleic acid intensities of XFC in leaves and roots under 4-day shading treatments displayed a upward trend compared to those of the control, but the intensities showed a clear downregulation under 12-day shading treatments. These results indicated that XRC leaves and roots under 4 days of shading treatment had a more active metabolism, which might promote the content of aroma compounds in tea plants (Li J. et al., 2019). In summary, it could be inferred that 4-day shading treatment seems to be advantageous for the aroma of XFC.

#### Flavonoid Metabolism

**Figure 9:**
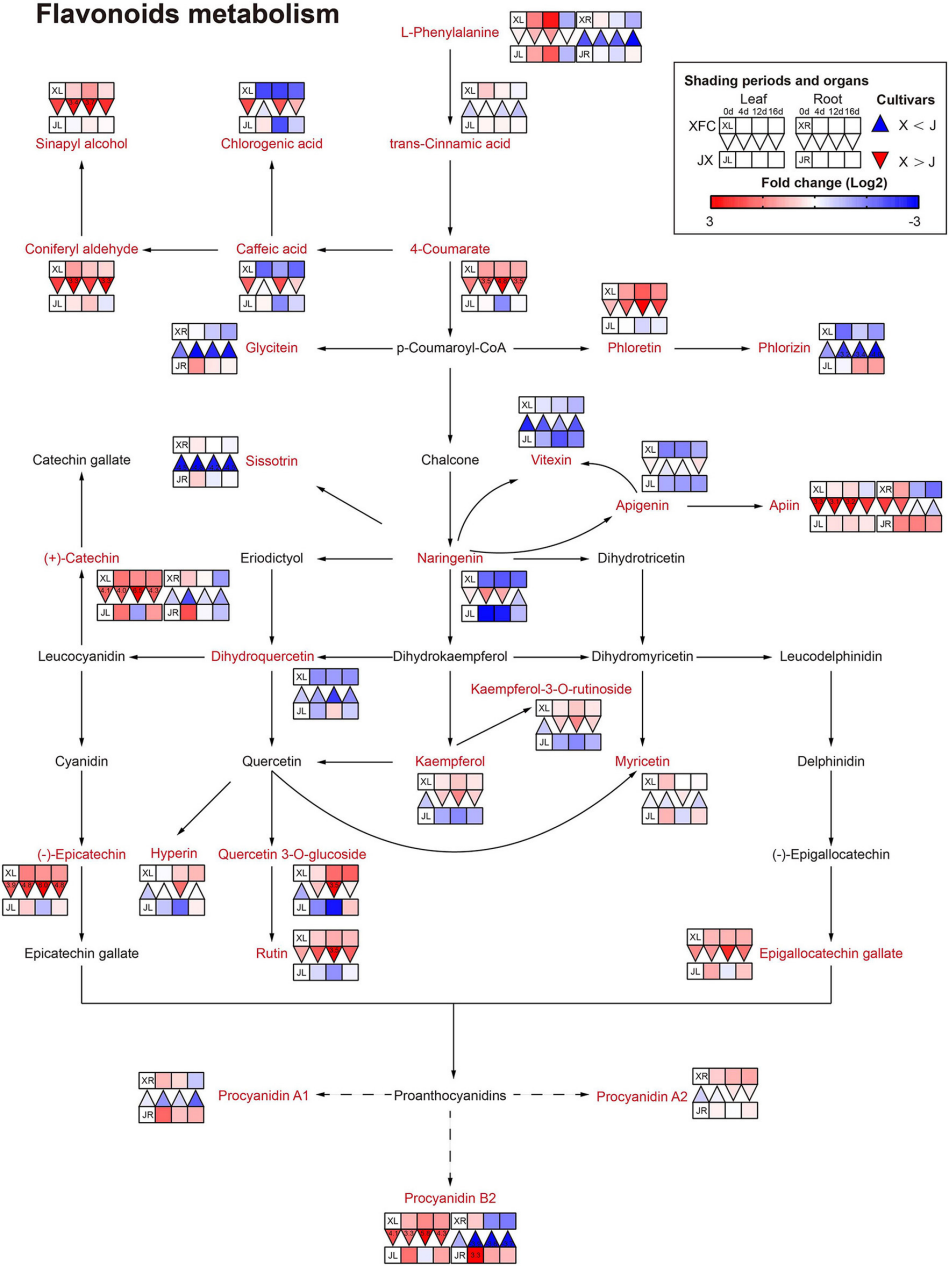
The metabolite differences during flavonoids metabolism in the two tea cultivars under different shading periods. The identified metabolites are marked with a red color. In the legend, the squares at two lines express the different cultivars, “Xiangfeicui” (XFC) and “Jinxuan” (JX). The squares at eight columns express the different shading periods. The squares in the left part represent the leaves and the right part represents the roots. Blue indicates relatively low and red indicates relatively high intensity. The red triangle indicates relatively higher intensity of metabolites in XFC (X) than in JX (J), and the inverted blue triangle indicates relatively lower intensity of metabolites in XFC (X) than in JX (J). The degree of change was described with the depth of color, and the depth of the colors is based on the log2-fold change value between the samples. XL, leaf of “Xiangfeicui”; XR, root of “Xiangfeicui”; JL, leaf of “Jinxuan”; JR, root of “Jinxuan”.

It is generally known that flavonoids are carbon-based secondary metabolites, and flavonoids play an important role in the taste of tea (Lin et al., 2021). Catechins are proven antioxidants and can improve stress resistance in plants (Chobot et al., 2009). Proanthocyanidins are formed by combining multiple catechins and also have strong antioxidant capacity (Ma et al., 2019). As shown in Figure 9, the levels of (+)-catechin, procyanidin B2, and procyanidin A1 in the leaves and roots of JX, significantly increased after 4-day shading treatment, and then decreased under 12-day shading, indicating that the tea plants might exhibit a stress response in the early stages of shading, which was different from other plant studies (Hussain et al., 2020). In addition, the 12-day shading treatment reduced the concentrations of most flavonoids in JXL, including 4-Coumarate, Naringenin, Vitexin, Apigenin, (+)-catechin, (–)-Epicatechin, Epigallocatechin gallate, Kaempferol, Rutin, and some flavonols, while these compounds had upward trends and higher levels in XFL under shading. This result revealed that the response of the flavonoid contents between varieties to shading was different. In contrast to the varieties with relatively high catechin contents, JX with relatively low catechin contents, was more susceptible to down-regulation caused by shading. The flavonoid metabolism is reportedly influenced by the TCA cycle and the biosynthesis of carbohydrates and amino acids (Araújo et al., 2014). Thus, the flavonoid metabolism is controlled in many ways, and the shading treatments could differentially regulate the intensity of flavonoid metabolism in tea plants.

#### Nitrogen Metabolism

**Figure 10:**
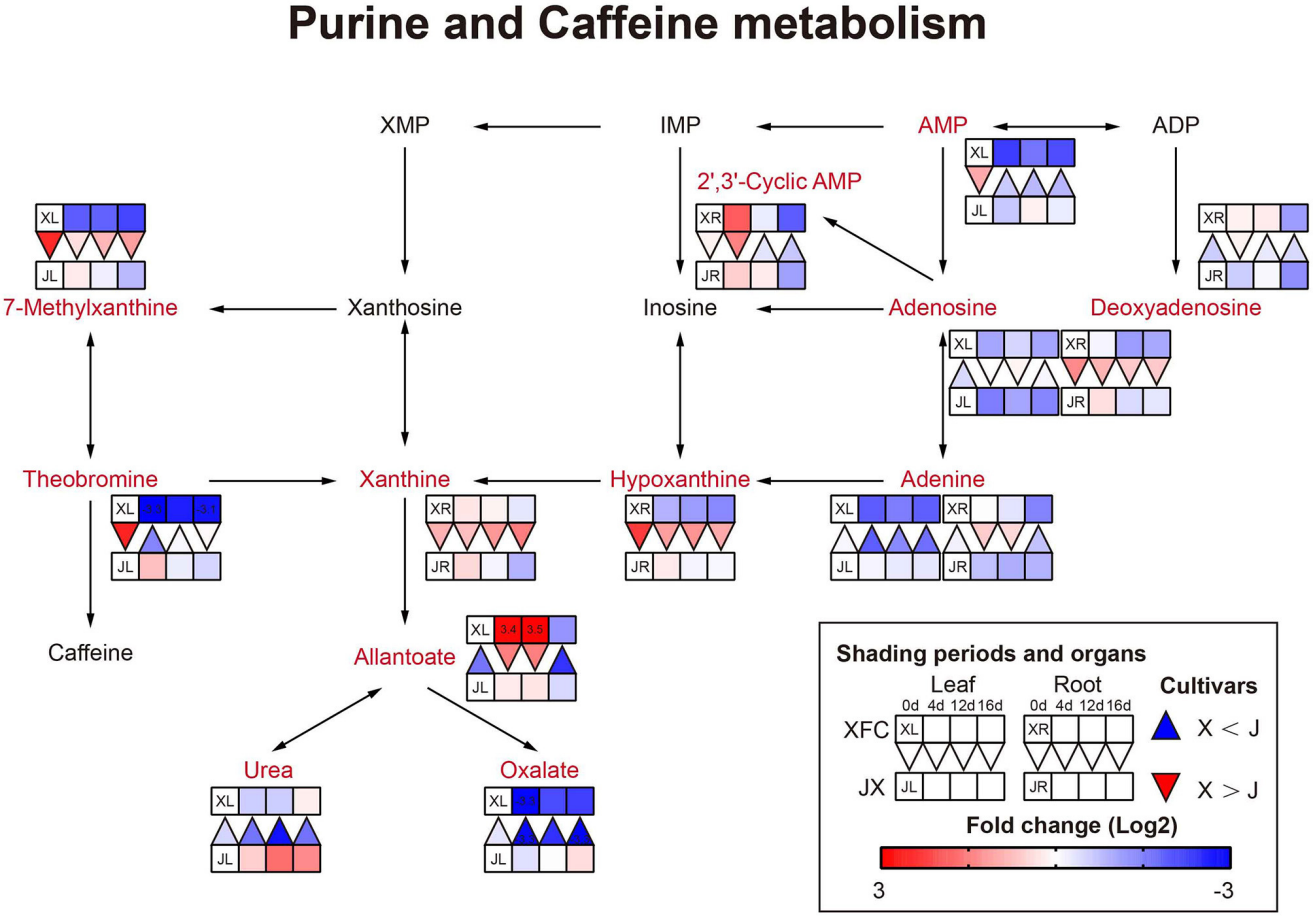
The metabolite differences during purine and caffeine metabolism in the two tea cultivars under different shading periods. The identified metabolites are marked with a red color. In the legend, the squares at two lines express the different cultivars, “Xiangfeicui” (XFC) and “Jinxuan” (JX). The squares at eight columns express the different shading periods. The squares in the left part represent the leaves and the right part represents the roots. Blue indicates relatively low and red indicates relatively high intensity. The red triangle indicates relatively higher intensity of metabolites in XFC (X) than in JX (J), and the inverted blue triangle indicates relatively lower intensity of metabolites in XFC (X) than in JX (J). The degree of change was described with the depth of color, and the depth of the colors is based on the log2-fold change value between the samples. XL, leaf of “Xiangfeicui”; XR, root of “Xiangfeicui”; JL, leaf of “Jinxuan”; JR, root of “Jinxuan”.

**Figure 11:**
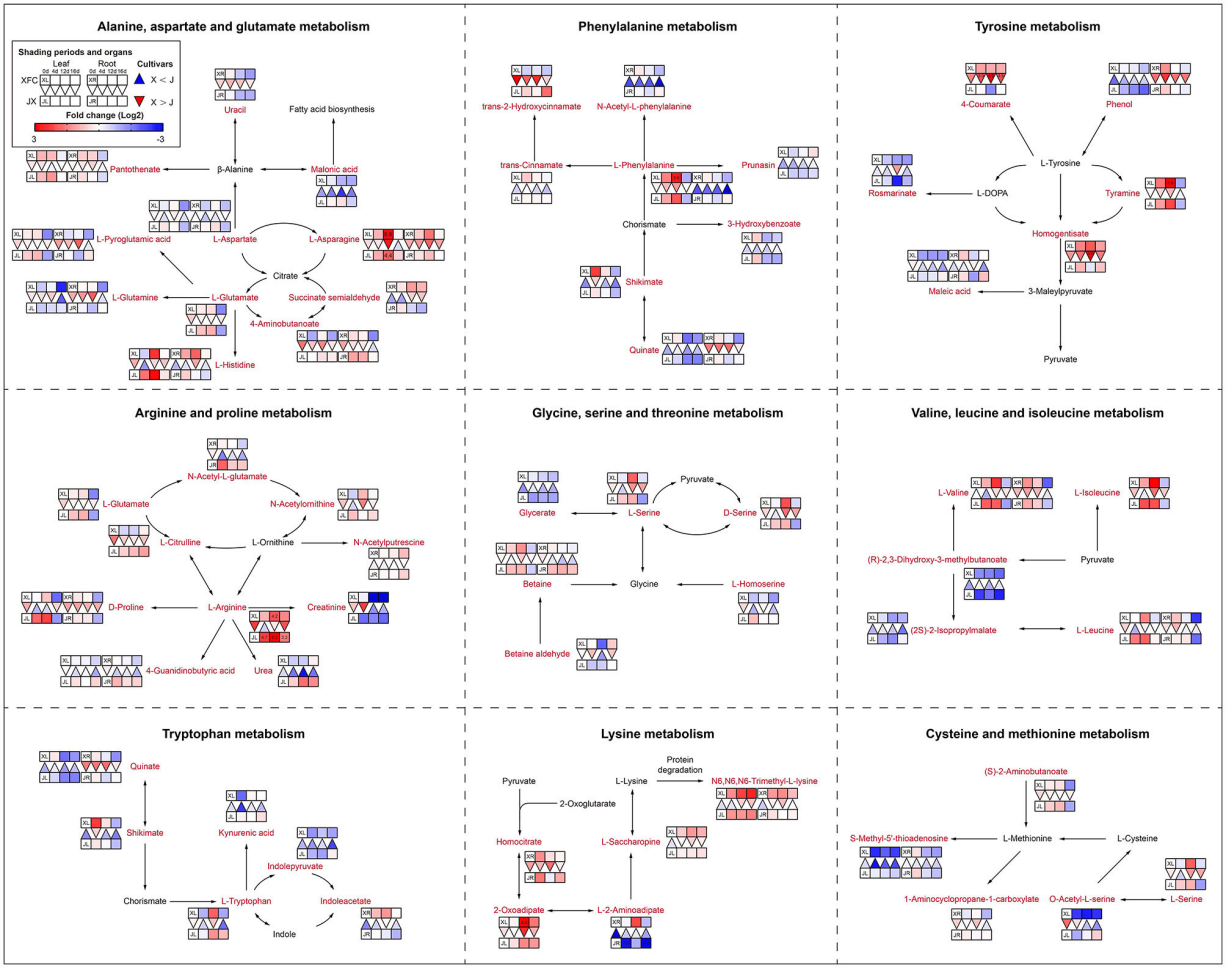
The metabolite differences during amino acid metabolism in the two tea cultivars under different shading periods. The identified metabolites are marked with a red color. In the legend, the squares at two lines express the different cultivars, “Xiangfeicui” (XFC) and “Jinxuan” (JX). The squares at eight columns express the different shading periods. The squares in the left part represent the leaves and the right part represents the roots. Blue indicates relatively low and red indicates relatively high intensity. The red triangle indicates relatively higher intensity of metabolites in XFC (X) than in JX (J), and the inverted blue triangle indicates relatively lower intensity of metabolites in XFC (X) than in JX (J). The degree of change was described with the depth of color, and the depth of the colors is based on the log2-fold change value between the samples. XL, leaf of “Xiangfeicui”; XR, root of “Xiangfeicui”; JL, leaf of “Jinxuan”; JR, root of “Jinxuan”.

Amino acids are main nitrogen-based metabolites that affect tea quality. The biosynthesis of amino acids occurs primarily in glycolysis, the TCA cycle and the oxidative pentose phosphate pathway (Fernie et al., 2004). The purine alkaloids were synthesized by the pentose phosphate pathway (Li Y. et al., 2016). As shown in Figure 10, purine and alkaloid compounds showed a significant decline under shading treatments in XFL, such as Adenine, 7-Methylxanthine, and Theobromine, while most metabolites were increased in shaded JXL, indicating that the shading was different for nitrogen allocation in different tea varieties (Li et al., 2020). It is possible that a small quantity of one substance at upstream results in a larger quantity accumulation of another downstream. As shown in Figure 11, there were nine pathways observed, which were associated with amino acids, such as alanine, aspartate and glutamate metabolism, phenylalanine metabolism and tyrosine metabolism. Nearly all the amino acids have a similar rule of change under shading treatments; the 4-day shading treatments increased the concentrations of amino acids in the tea leaves and roots, and the 12-day shading treatments led their concentrations to the highest levels, including L-Asparagine, L-Glutamate, L-Histidine, L-Phenylalanine, L-Arginine, L-Serine, L-Valine, and Betaine (Lee et al., 2013). However, the level of L-Glutamine, L-Aspartate, 4-Aminobutanoate, and L-Tryptophan were no significant change in 4-day shaded leaves. Meanwhile, our results displayed a significant decrease on the amino acid content after the relight treatment. Shading promotes nitrogen metabolism in tea leaves possibly by increasing proteolysis, and in the present study, we found that this increase occurred in both leaves and roots (Yu and Yang, 2020; Yang et al., 2021). These findings suggested that the longer shading treatments was more conductive to N metabolism both in the tea leaves and roots, which was the primary reason that why shaded green tea was fresher than the regular tea (Zhang Y. et al., 2021). This was inconsistent with the conclusion of some previous studies (Li et al., 2020). In general, the N provision for sustaining growth is contributed by the translocation of soil N from new uptake by the root and the remobilization of the N reserves stored within the plants (Yang et al., 2020). In the present study, the increased content of nitrogen-based metabolites in roots might indicate that shading seems to promote nitrogen uptake from the soil in tea plants (Li et al., 2021).

## Conclusion

Through physiological and biochemical analysis, phenotypic analysis, untargeted metabolomics methods, combined with the expression analysis of selected genes, we have found that long-term shading can improve the quality of summer and autumn green tea compared with short-term shading. Short-term shading may have caused stress on tea plants. By using metabolomics analysis of leaves and roots, we found that carbon and nitrogen metabolism jointly promoted tea quality, including sugar metabolism, lipid metabolism, flavonoid metabolism, amino acid metabolism, and purine alkaloid metabolism. Our work shows that metabolites in different tea varieties, including green tea quality-related compounds like amino acid and catechins, are affected differently during different shading periods. A limitation of this study is that we did not explore the effect of shading on the tea processing. Hence, considerably more work will need to be done to determine which metabolites and genes regulate the quality of shaded tea during processing.

## Data Availability Statement

The original contributions presented in the study are included in the article/Supplementary Material, further inquiries can be directed to the corresponding author/s.

## Author Contributions

CSha: conceptualization, writing—original draft, writing—review, editing, and mapping. HJ and JiahC: investigation, resources, and supervision. CZ and JL: validation. JianC: formal analysis. YL: data curation. JH and BY: visualization. ZL: project administration. CShe: project administration and funding acquisition. All authors contributed to the article and approved the submitted version.

## Funding

This work was supported by Joint Funds of National Natural Science Foundation of China (Grant No. U19A2030); the National Natural Science Foundation of China (Grant No. 31271789); Central Committee Guides Local Science and Technology Development Program (Grant No. 2019XF5041); and the Special Project for the Construction of Modern Agricultural Industrial Technology Systems in Hunan Province (Xiang Nongfa) (Grant No. 2020112).

## Conflict of Interest

The authors declare that the research was conducted in the absence of any commercial or financial relationships that could be construed as a potential conflict of interest.

## Publisher's Note

All claims expressed in this article are solely those of the authors and do not necessarily represent those of their affiliated organizations, or those of the publisher, the editors and the reviewers. Any product that may be evaluated in this article, or claim that may be made by its manufacturer, is not guaranteed or endorsed by the publisher.
