# Supplementary material for: Carbon and Nitrogen Metabolism Are Jointly Regulated During Shading in Roots and Leaves of Camellia Sinensis
Source: Front Plant Sci. 2022 Apr 15;13:894840. doi: 10.3389/fpls.2022.894840 (PMC9051521; doi:10.3389/fpls.2022.894840)
Supplement: Supplementary file 2 [file Table_2.DOCX]

Supplementary Material

**Supplementary Table 1.** Definition and scale for sensory characteristics of green tea.

| Factors | Description | Scores | Score coefficient |
| --- | --- | --- | --- |
| Appearance | Tender, tip, much evenly, neat, bloom and even colour | 90-99 | 25% |
|  | Little tender, less tip, evenly, little neat, bloom | 80-89 |  |
|  | Less tender, regged, dull dry | 70-79 |  |
| Brewing color | Clear, bright (differences between categories) | 90-99 | 10% |
|  | Little bright (differences between categories) | 80-89 |  |
|  | Less bright, suspension (differences between categories) | 70-79 |  |
| Aroma | High aroma, fragrance, pure and normal, tender flavour | 90-99 | 25% |
|  | Little fragrance, normal | 80-89 |  |
|  | Sulky odour, over fired aroma, grass odour | 70-79 |  |
| Taste | Mellow and thick, fresh, heavy and mellow, sweet | 90-99 | 30% |
|  | Heavy and strong, little mellow | 80-89 |  |
|  | Less mellow, heavy and astringency, grassy | 70-79 |  |
| Infused leaf | Fine and tender, bright, tender and even | 90-99 | 10% |
|  | Little bright, even, soft | 80-89 |  |
|  | Uneven, less tender, hard and mixed | 70-79 |  |

Note: To rank different levels of green tea, the characteristics were divided to three grades: 90-99, 80-89 and 70-79. This evaluation standard refers to the Chinese standards of GB/T 23776-2018 “Methodology for sensory evaluation of tea”.

**Supplementary Table 2.** Primer sequences for the quantitative RT-PCR of genes.

| No. | Gene Symbol | GeneBank | Forward primer | Reverse primer |
| --- | --- | --- | --- | --- |
| 1 | PAL | KF303590 | CGGTGAGCCAAGTAGCGAAGC | ACGGTCTACCACTCTGAGCAAGTC |
| 2 | C4H | AY641731 | CGCCGTAGTCCTTGCCATCAC | ACTTGGAGCCAGTTGCCGAATAC |
| 3 | 4CL | MN879747 | CGTGGTCCTCAAATTATG | CATCATCGTCGTCTACAT |
| 4 | CHS | AY656677 | TTACTAATAGCGAGCATAAGGT | CTAGCATCAAGCGAAGGT |
| 5 | CHI | DQ904329 | GTGATGGATGAAGTTGTG | AAGAGAGAAAGCAGAGTC |
| 6 | F3H | AY641730 | GCGGATCATCAGGCAGTAGTGAAC | ATCGGCTTCTCTCCCTCCCTAATC |
| 7 | F3’H | KY615695 | ATCTGCTCCGTCCATCTCTTCTCC | CTAGGTTCACTGCTGCCGCTTG |
| 8 | F3’5’H | AY945842 | AATCCTGGTGAAGAGAAG | TCTATTATGCTTGATGATGTG |
| 9 | FLS | DQ198089 | GGAGAACAGCAAGGATATCG | TCTCCTCCTGTGGGAGCTTA |
| 10 | DFR | AB018685 | AGTTGTGTCGTTCTCATC | GTATCAATGGCTCCTCTG |
| 11 | ANS | AY830416 | TAATGGCAAGTACAAGAG | CAATGGCTTCAAGATAATC |
| 12 | ANR | AY641729 | GCGAAGTTGATCCTCTCGTC | AACCACATCGTCAAGTGAACA |
| 13 | LAR | AY169404 | GGGGCATCCTGTATCAAAGA | CCGCATACCTTTCAGTCCAT |
| 14 | CsGS1.1 | MG778703 | CAGCACCAAGTCTACGAGGA | AATCATGGAAGTAACCACA |
| 15 | CsGS2 | MG778706 | CTGGAACGGTGCAGGATGC | GCCCCACGCGGATTGAACA |
| 16 | CsTSΙ (in root) | TEA015198.1 | GTTGATGTTTCTGGGCAGCA | CTCACCCACACCAGTCAGAT |
| 17 | GAPDH | GE651107 | TTGGCATCGTTGAGGGTCT | CAGTGGGAACACGGAAAGC |

**Supplementary Table 3.** Sensory quality scores of the green tea samples.

| Shading period | Appearance | Brew color | Aroma | Taste | Infused leaf | Total score |
| --- | --- | --- | --- | --- | --- | --- |
| 0XFC | 89.8±0.3c | 92.7±0.3a | 88.6±0.9e | 89.3±0.4e | 85.0±0.4c | 89.2±0.2e |
| 4XFC | 90.9±0.2b | 91.8±0.3b | 89.4±0.2d | 90.6±0.8d | 86.5±0.4b | 90.1±0.2d |
| 12XFC | 91.4±0.4a | 91.4±0.2c | 90.0±0.4d | 91.3±0.8cd | 87.9±0.9a | 90.7±0.2c |
| 0JX | 89.7±0.4c | 90.8±0.3d | 92.0±0.4c | 91.5±0.5c | 84.0±1.1d | 90.4±0.2d |
| 4JX | 90.1±0.2c | 90.6±0.2d | 93.3±0.8b | 92.4±0.5b | 86.9±0.7b | 91.3±0.3b |
| 12JX | 90.9±0.2b | 90.1±0.2e | 94.7±0.4a | 94.1±0.5a | 88.0±0.4a | 92.4±0.2a |

Note: Data were presented as mean ± SD (standard deviation) and were assessed by one-way ANOVA followed by Duncan’s multiple range test. 0XFC, unshaded green tea of ‘Xiangfeicui’ cultivar (XFC); 4XFC: shaded green tea of XFC after 4 days of shading; 12XFC: shaded green tea of XFC after 12 days of shading; 0JX: unshaded green tea of ‘Jinxuan’ cultivar (JX); 4JX: shaded green tea of JX after 4 days of shading; 12JX: shaded green tea of JX after 12 days of shading.


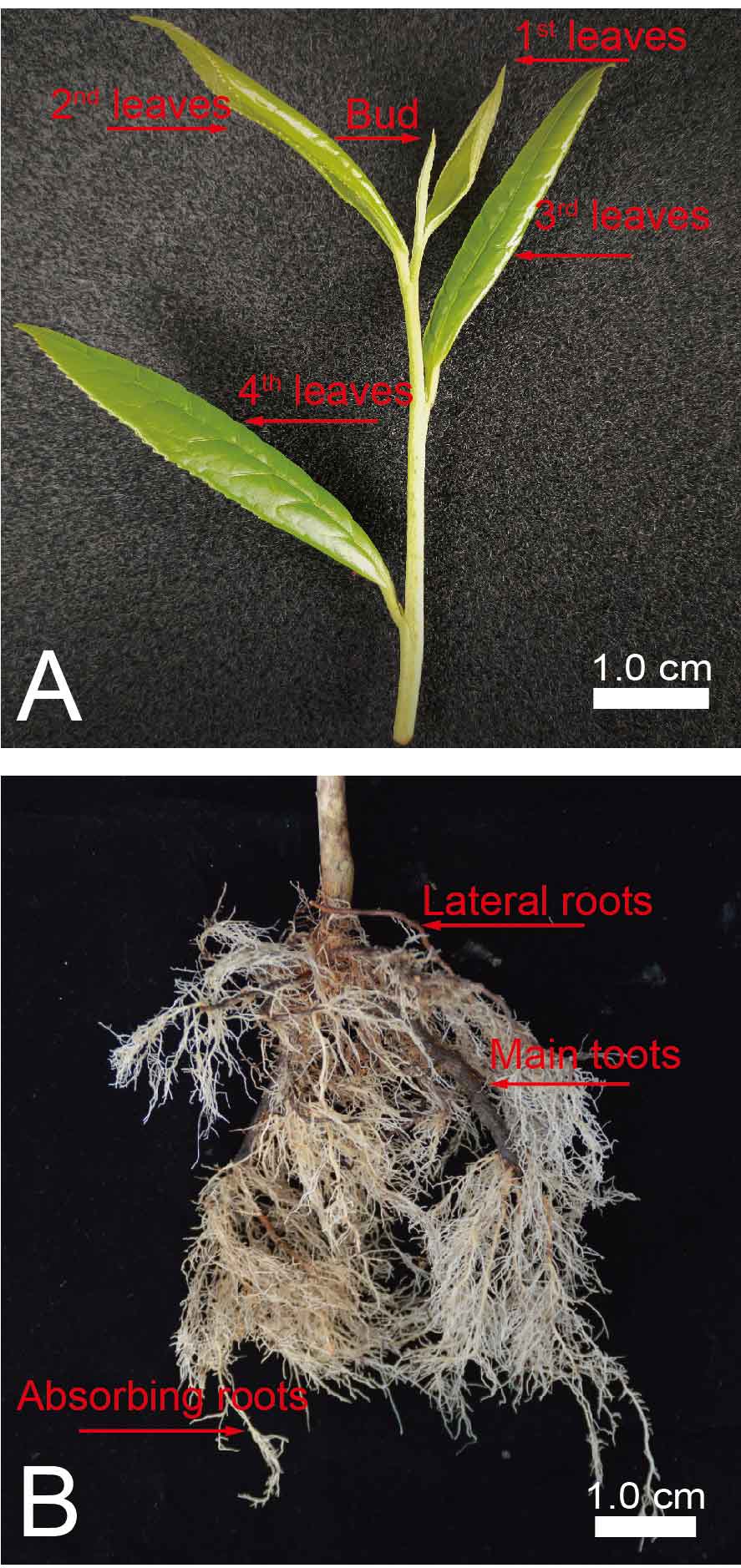


**Supplementary Figure 1.** Different positions of the tea leaves and tea roots.


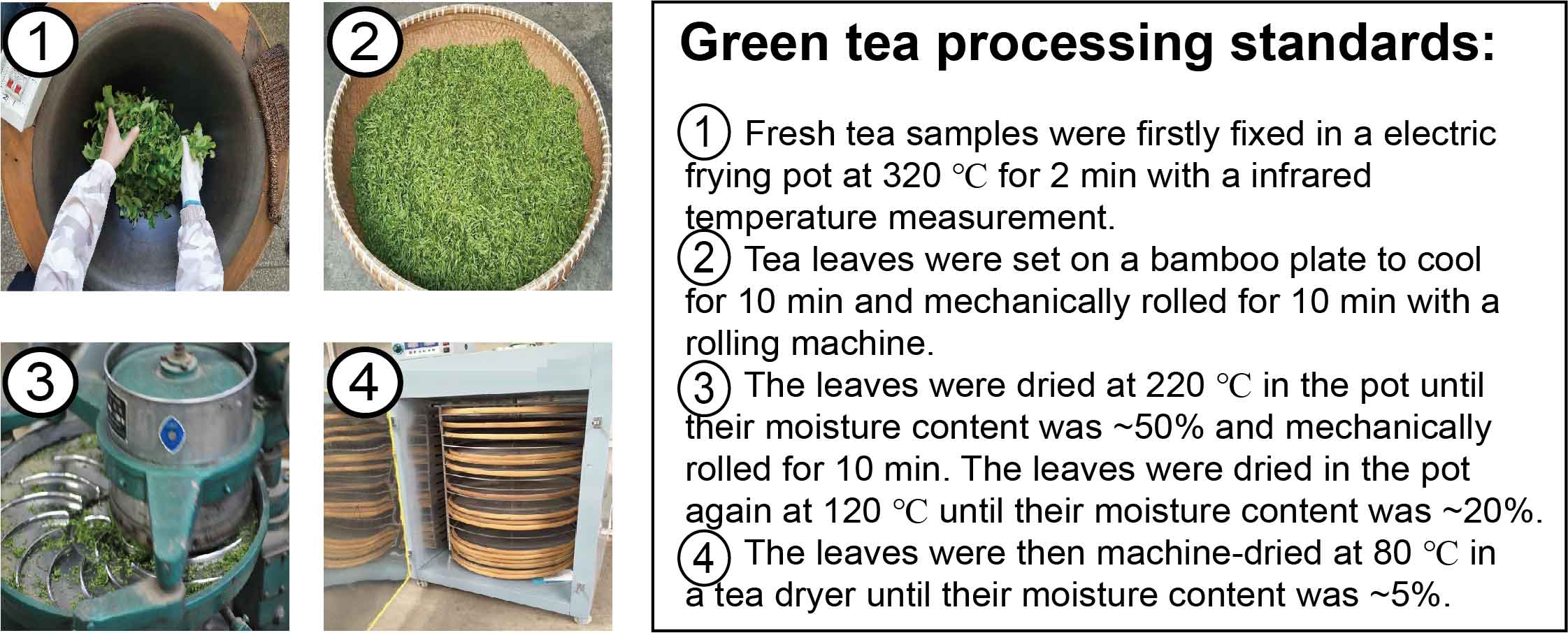


## Supplementary Figure 2. Green tea processing standards.


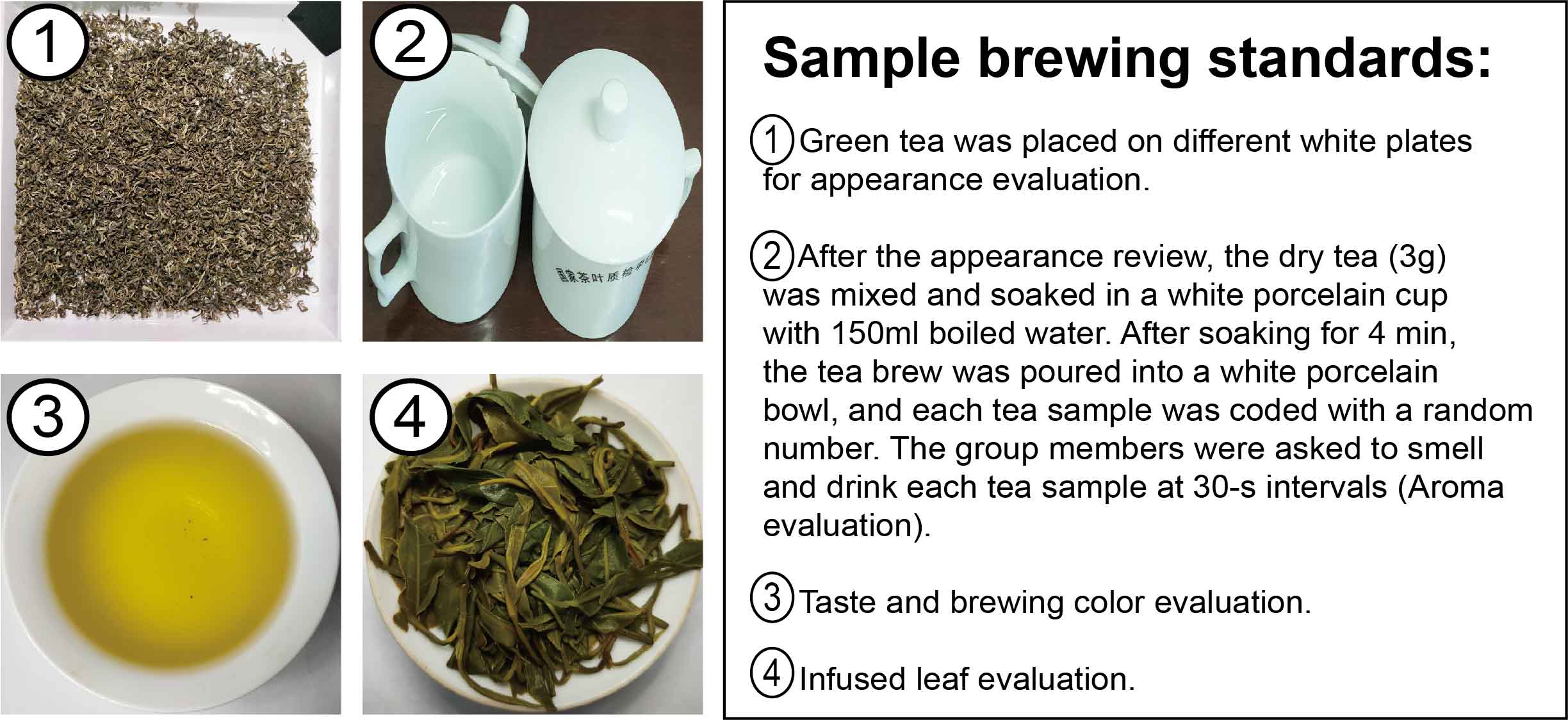


## Supplementary Figure 3. Sample brewing standards.
